# Network and Systems Medicine: Position Paper of the European Collaboration on Science and Technology Action on Open Multiscale Systems Medicine

**DOI:** 10.1089/nsm.2020.0004

**Published:** 2020-07-06

**Authors:** Blandine Comte, Jan Baumbach, Arriel Benis, José Basílio, Nataša Debeljak, Åsmund Flobak, Christian Franken, Nissim Harel, Feng He, Martin Kuiper, Juan Albino Méndez Pérez, Estelle Pujos-Guillot, Tadeja Režen, Damjana Rozman, Johannes A. Schmid, Jeanesse Scerri, Paolo Tieri, Kristel Van Steen, Sona Vasudevan, Steven Watterson, Harald H.H.W. Schmidt

**Affiliations:** ^1^ Plateforme d'Exploration du Métabolisme, MetaboHUB Clermont, Université Clermont Auvergne, INRAE, UNH, Clermont-Ferrand, France.; ^2^ TUM School of Life Sciences Weihenstephan (WZW), Technical University of Munich (TUM), Freising-Weihenstephan, Germany.; ^3^ Holon Institute of Technology, Holon, Israel.; ^4^ Institute of Vascular Biology and Thrombosis Research, Center for Physiology and Pharmacology, Medical University of Vienna, Vienna, Austria.; ^5^ Medical Centre for Molecular Biology, Institute of Biochemistry, Faculty of Medicine, University of Ljubljana, Ljubljana, Slovenia.; ^6^ Department of Clinical and Molecular Medicine, Norwegian University of Science and Technology, Trondheim, Norway.; ^7^ The Cancer Clinic, St. Olav's University Hospital, Trondheim, Norway.; ^8^ Digital Health Systems, Einsingen, Germany.; ^9^ Department of Pharmacology and Personalised Medicine, Faculty of Health, Medicine and Life Science, Maastricht University, Maastricht, The Netherlands.; ^10^ Department of Infection and Immunity, Luxembourg Institute of Health, Esch-sur-Alzette, Luxembourg.; ^11^ Institute of Medical Microbiology, University Hospital Essen, University of Duisburg-Essen, Essen, Germany.; ^12^ Department of Biology, Faculty of Natural Sciences, Norwegian University of Science and Technology, Trondheim, Norway.; ^13^ Department of Computer Science and Systems Engineering, Universidad de La Laguna, Tenerife, Spain.; ^14^ Centre for Functional Genomics and Bio-Chips, Institute of Biochemistry, Faculty of Medicine, University of Ljubljana, Ljubljana, Slovenia.; ^15^ Department of Physiology and Biochemistry, Faculty of Medicine and Surgery, University of Malta, Msida, Malta.; ^16^ CNR National Research Council, IAC Institute for Applied Computing, Rome, Italy.; ^17^ GIGA-R Medical Genomics-BIO3, University of Liège, Liège, Belgium.; ^18^ Georgetown University Medical Centre, Washington, District of Columbia, USA.; ^19^ Northern Ireland Centre for Stratified Medicine, Ulster University, Londonderry, United Kingdom.; ^20^ Department of Pharmacology and Personalised Medicine, Faculty of Health, Medicine and Life Science, MeHNS, Maastricht University, The Netherlands.

**Keywords:** big data, data integration, integrated health care, omics, systems medicine

## Abstract

**Introduction:** Network and systems medicine has rapidly evolved over the past decade, thanks to computational and integrative tools, which stem in part from systems biology. However, major challenges and hurdles are still present regarding validation and translation into clinical application and decision making for precision medicine.

**Methods:** In this context, the Collaboration on Science and Technology Action on Open Multiscale Systems Medicine (OpenMultiMed) reviewed the available advanced technologies for multidimensional data generation and integration in an open-science approach as well as key clinical applications of network and systems medicine and the main issues and opportunities for the future.

**Results:** The development of multi-omic approaches as well as new digital tools provides a unique opportunity to explore complex biological systems and networks at different scales. Moreover, the application of findable, applicable, interoperable, and reusable principles and the adoption of standards increases data availability and sharing for multiscale integration and interpretation. These innovations have led to the first clinical applications of network and systems medicine, particularly in the field of personalized therapy and drug dosing. Enlarging network and systems medicine application would now imply to increase patient engagement and health care providers as well as to educate the novel generations of medical doctors and biomedical researchers to shift the current organ- and symptom-based medical concepts toward network- and systems-based ones for more precise diagnoses, interventions, and ideally prevention.

**Conclusion:** In this dynamic setting, the health care system will also have to evolve, if not revolutionize, in terms of organization and management.

## Introduction

### Why we need new medicine

**FIG. 1.**
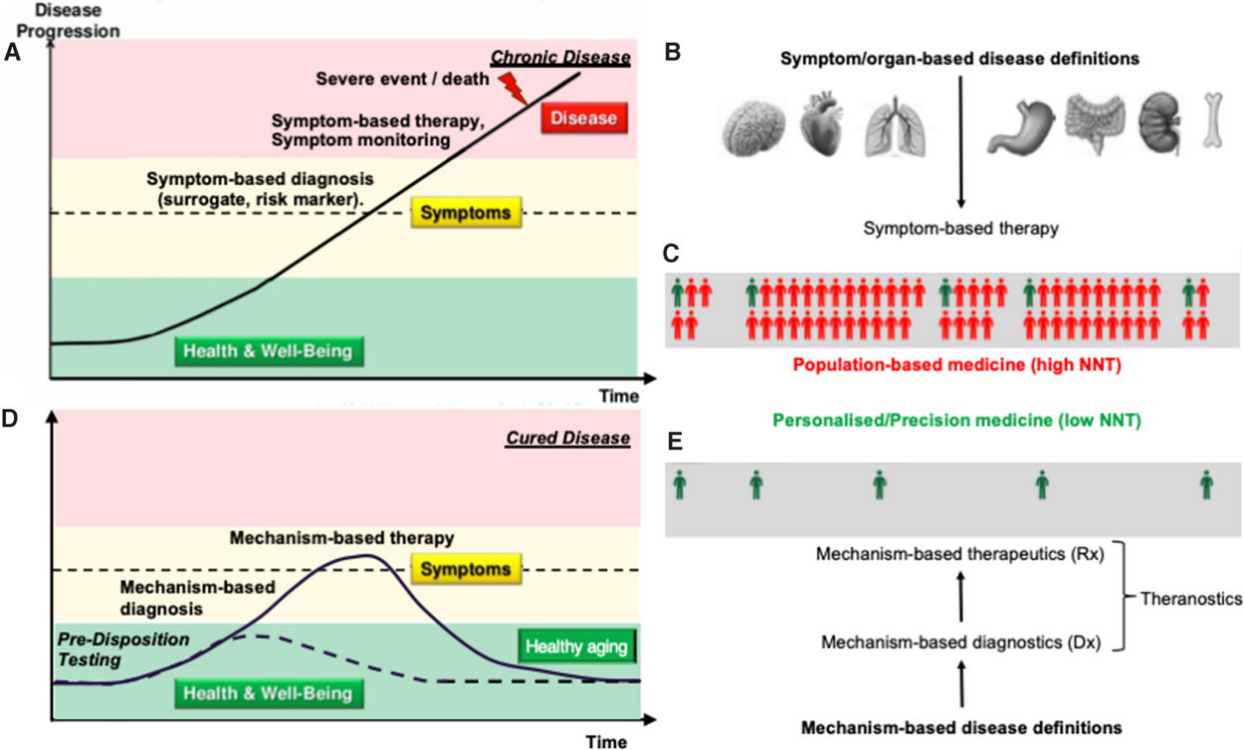
Medical knowledge gaps and the ground-breaking nature of Network and Systems Medicine. **(A)** Time course of most chronic diseases without knowing the causal mechanism. Diagnosis relies on signs and symptoms pointing to **(B)** specific organs. Therapy focuses on achieving patient-relevant outcomes only in **(C)** a small fraction (green) of patients.^5^ **(D)** Network and Systems Medicine aims at defining and diagnosing a disease mechanistically, and at treating it with higher precision, based on **(E)** mechanism-based diagnostics and therapeutics (i.e., theranostics).

About 70% of all medical interventions pertain to the prescription of a drug. However, for several drugs that are on the market, population-based studies fail to show patient-relevant benefits.^1,2^ Research covering drug approval since the 1970s suggests that only a limited number of new drugs provide real advances over existing ones; most studies place the proportion of true innovation at less than 15%.^3^ For every person they do help, the 10 highest grossing drugs in the United States fail to improve the conditions of most other patients, leading to so-called high “numbers needed to treat” (NNT, Fig. 1C).^4^

Since the 1950s, the efficacy of translating biomedical research into successful drug discovery is on a constant decline.^4,5^ Two key factors have contributed to this innovation roadblock. One is the irreproducibility of pre-clinical and basic research data^6^ to which, besides data manipulation/fabrication, poor study quality, such as lack of statistical power, and positive publication bias by scientific journals are the main contributors.^7,8^ The second reason is the conceptual medical knowledge gap about many of our current, in particular chronic, disease definitions. Except for infectious and rare diseases, most disease definitions are based on signs and reported symptoms, pointing to organ-centric mechanisms (Fig. 1B), and not on causal molecular mechanisms that, for many diseases, are not even known. Consequently, there is a disease-based care system, where the focus is placed on treating and monitoring the symptoms (Fig. 1A), rather than true health care that treats the causes by helping to implement lifestyle decisions for a healthier life.

A drug can only be effectively developed and applied in a precise manner if the molecular disease mechanism is known. Not knowing a disease mechanism also affects basic and pre-clinical (animal) research, where often cellular or animal models that mimic symptoms of human disease are used, but neither the underlying mechanism of the animal model nor that of the human disease is known.^9–14^ Noteworthy exceptions to these limitations and shortcomings are again infectious or rare diseases, where a precise—often monogenetic—mechanism is known. Many common and complex clinical disease phenotypes, once they are fully endo-phenotyped and mechanistically understood, will segregate into several distinct mechanotypes.^15^

Many common diseases appear complex, because we combine several molecular diseases under one umbrella term based on shared prominent signs and symptoms. For example, high blood pressure is in 95% of the cases diagnosed as essential hypertension, meaning that the blood pressure is elevated, but we do not know why. These patients are then treated with different blood vessel-dilating drugs and the clinical sign, elevated blood pressure, disappears, yet the cause is not known and remains untreated. Once mechanistically understood, chronic diseases can be cured or even prevented and no longer just treated (Fig. 1D).

The United Kingdom National Institute for Health and Care Excellence published a list of the absolute benefits of treatments of common conditions in terms of their NNT. For example, for every thousand low-risk patients prescribed statins for primary prevention, only a single stroke is prevented per year; one needs to treat more than a thousand patients with antihypertensives per year to prevent one death, more than 800 to prevent one heart attack, and nearly 600 to prevent one stroke. In high-risk patients, the NNTs are smaller, but the problem persists.^16^ Thus, a move toward a more precise ideally curative therapy that works for almost every patient is of utmost importance.

### Applied biomedical research and drug discovery

This efficacy problem also pertains to basic research and its translation into applications such as drug discovery. Of 25,190 articles published from 1979 to 1983 in the six basic science journals, *Science, Nature*, *Cell*, the *Journal of Experimental Medicine*, and the *Journal of Clinical Investigation*, which had the highest impact factors in 2000, and the *Journal of Biological Chemistry*, which received the most citations, only a single claim of relevance may have led to actual application follow-up.^17^ Regarding the discovery of new drugs, for three decades, costs have increased exponentially and are now stable at an extremely high level. Since the 1950s, however, the efficacy of drug discovery is on a constant logarithmic decline, indicating a fundamental and conceptual problem of how we define and approach disease.^5^ For drug discovery, recently, systematic drug repurposing is being increasingly explored and represents a conceptual change to a mechanism-based disease definition, allowing for a mechanism-based patient stratification, which increases the precision for any subsequent mechanism-based drug intervention. This will massively de-risk drug development, yet at the downside that in the future, drugs will be developed for much smaller patient numbers.

### From single targets to validated, causal networks

In the diseasome, disease clusters are mechanistically defined by several genes and proteins forming a signaling network.^18^ This has been most extensively hypothesized for three distinct networks for macular degeneration^18,19^ and cancer.^20^ The validity of these networks is essential, because both the diagnostic and therapeutic strategies reside on it. Defining the causal signaling network is not trivial and not at all obvious. It is state-of-the-art to rely on highly curated signaling pathway databases such as the Kyoto Encyclopedia of Genes and Genomes (KEGG) or WikiPathways, a collection of manually drawn pathway maps representing our apparent knowledge on molecular interactions, reactions and relation networks, or review articles. KEGG, however, shows 29 cyclic guanosine monophosphate (GMP) and 12 reactive oxygen pathways, none of which is comprehensive and all of which fail to cover a recently discovered functional and molecular link between the two,^21^ uniting both, in fact, to one network. Moreover, subcellular compartmentalization and transition over time also matter in defining disease modules,^18,21^ contributing to further deviation from static pathway concepts.

### Mechanism-based diagnostics

Biomarkers are increasingly hypothesized as important for precision medicine,^22–24^ although the term is ambiguous and used for several applications such as screening, stratification, efficacy, differentiation, toxicity, and prognosis. In place of validated causal disease mechanisms, the state-of-the-art biomarkers used for these purposes are mostly correlative surrogate and omic markers, rarely established risk factors, and never a full functional analysis of a patient's activity state of a causal disease mechanism.^22^ The limited availability of predictive and precise biomarkers represents a key bottleneck in the progress from mechanism-based disease definition to clinical validation by mechanism-based therapeutic intervention. This causes inefficient drug therapy and clinical trials with a high failure rate (see above).

### From single or combination therapy to network pharmacology

The first line of treatment for many diseases involves the administration of a single drug, assuming a single relevant target. If the therapeutic effect is insufficient, drugs are combined. Sometimes, such combination therapies can get out of control when so-called poly-pharmacy results in 10 or more drugs being prescribed to a patient with unwanted drug–drug interactions and side effects. Network pharmacology may be easily confused with such combination therapies. The important difference, however, is that in combination therapy symptomatically acting drugs on unrelated targets are combined and act in an additive manner; whereas in network pharmacology, all drugs act on the same and causal network, and are thereby highly synergistic. This allows for a substantial reduction in the dose of each drug while still achieving the same therapeutic effect. This will in all likelihood reduce any side effect of each drug or possible unwanted drug–drug interactions.^21,25^

### Definition and goals of network and systems medicine

Major socioeconomic innovations are not only triggered by unmet needs as described earlier but also by critical technological advances. Insofar, network and systems medicine would not have emerged without decades of development of its antecedent discipline, that is, systems biology as defined by pioneers in the field.^26,27^ Therefore, it is impossible to discuss systems medicine alone without first talking about systems biology.

Systems biology emphasizes analyzing interactions within complex biological systems by using holistic and integrative high-throughput experimental and computational approaches. One of the hallmarks in the complex systems, such as multicellular organisms and multiorgan organisms (e.g., animals and humans), is that several components (different cell types, tissues, or organs) interact with each other as a local subnetwork or global network to generate emergent effects.^28,29^ The challenges behind this hallmark cannot be solved *per se* by the reductionist paradigm that decomposes the complex systems into smaller and simpler components and understands their functions and roles one by one. Thus, the emergence of systems biology is to tackle the essential limits of the reductionist approaches.^30^

Systems biology focuses on basic mechanisms and principles in biology or on most translational preclinical research, but systems medicine aims at directly handling the challenges related to health and diseases.^31–33^ In a way, it can also be considered as a modern advancement of physiology. So far, there is no consensus on the definition of systems medicine, an emergent and fast evolving field. Our perception about systems medicine is the application of systems biology approaches to the clinical settings of individuals by the combination of large-scale, multilayer, high-throughput quantitative molecular and image measurements at different spatial scales (from molecules, through cells and tissues, to organs), over various time scales, with different types of clinical information.^34^

The aims of systems medicine are also multidimensional, that is, from the understanding of disease mechanisms to accurate diagnosis, prediction, and eventual prevention by using accessible biopsies, tissues, and samples, to patient subgroup stratification of complex diseases, to the development of novel approaches in drug discoveries, to more precision treatment based on tailored measurements of distinct patients.^35^ Systems medicine is based on a holistic approach to medicine, in opposition to the current symptom/organ-based view. As proposed by Leroy Hood, systems medicine should eventually enable predictive, preventive, personalized, and participatory (P4) medicine to improve the wellness of our society.^36^ Network and systems medicine is at the crossroad of pure and applied sciences, wet and dry labs, life, and computer sciences. Main scientific and technological components of this new field have, therefore, yet to emerge and evolve into a well-established process.

Network and systems medicine is built similarly to a modern knowledge discovery flow. To implement systems medicine, as the name “systems” indicated, the first required technology should be the development of system-level multidimensional technologies. In this context, the present review on systems medicine first introduces the intended outcomes and definitions. It discusses in the “Basic science and data for network and systems medicine” section the required technologies for multidimensional data generation, the current data availability, as well as the computational tools for data integration and interpretation. In the “Clinical Applications” section, it illustrates its potentiality through some clinical applications. Finally, it discusses the current remaining issues and prospects of this large domain.

## Basic Science and Data for Network and Systems Medicine

Network and systems medicine is built similarly to a modern knowledge discovery flow. In particular, multidimensional omics data generation and integration are key elements in the big data analytics era.

### Multidimensional omics

The importance of taking into account the complexity of biological systems has been recognized as the basis of systems approaches. Indeed, it appears that in individuals, a different combination of genetic and environmental factors defines the pathology progress, which accumulates with age. We face co-occurrence of pathologies in the ever-aging population. In addition to cardiovascular complications, there is a rise in neurodegenerative pathologies and metabolic pathologies, where diabetes mellitus and nonalcoholic fatty liver disease are among the key components. It is important to note that metabolic diseases (metabolic syndrome, type 2 diabetes, osteoporosis, etc.) show strong comorbidities or co-occurrence with other diseases, such as cardiovascular diseases, cancers, and even neurodegenerative diseases, all major health problems of today's societies.^37–39^

There is a challenging situation where, on one hand, there is a large progress in understanding the molecular players of disease stages and overlap with other diseases; whereas on the other hand, the inconsistencies from different studies and different populations leave the impression that we are indeed at the start. In this context, the objective of omics research within systems medicine is to study and understand regulatory mechanisms, identify corresponding specific biomarkers, and characterize their interaction within and between systems,^40^ with the analysis of large sets of biological molecules, including genomics, epigenomics, proteomics, metabolomics, and much more, in combination with methodologies from the computer and mathematical science.

Genome-wide association studies (GWASs) have shown their importance in the discovery of single-nucleotide polymorphisms as markers associated with disease-specific clinical phenotypes or their risk factors. As an example, in liver diseases, the genome-wide association studies, transcriptome analyses, meta-analyses, and other clinical studies in different populations and ethnic backgrounds are until 2019 concordant in polymorphisms of a single gene, *PNPLA*3.^41^ However, liver pathologies remain a major health burden of modern societies where sex dimorphism remains a crucial, yet neglected factor.^42,43^

Generally speaking, complex disease phenotypes can rarely be explained by a single gene, and genomic analysis integrated with protein–protein interaction networks has evidenced the role of groups of genes and variants, and new pathways in multiple diseases.^44^ Therefore, the need for new disease risk models has emerged, including not only genetic factors, transcripts, and proteins but also elements such as metabolites, with the metabolome being closer to the phenotype.

Metabolomics, described as a global analysis of small molecules present in a biofluid (blood, urine, saliva, etc.), produced or modified as a result of stimuli (intervention, drug, genetic perturbations, etc.),^45,46^ is giving an integrated view of metabolism. Among different approaches, the untargeted strategy is a data-driven approach dedicated to biomarker discovery. Based on the use of multiple analytical platforms, such as mass spectrometry, it allows the detection of thousands of features and offers the possibility of characterizing global alterations associated with disease conditions.^47^ It has been widely applied in epidemiology for metabolic disease diagnosis and candidate biomarker discovery, pathophysiological exploration of underlying mechanisms, and diagnosis and prognosis.^48,49^ It is now recognized as a powerful phenotyping tool to better understand not only the biological mechanisms involved in pathophysiological processes but also the complexity of regulations in interaction with environmental factors.

The concept of the exposome was defined to characterize the environmental exposure in a broad sense of “non-genetic” factors, considering internal, specific external, and general external exposure.^50^ In particular, important advances have also been done for the identification of the contribution of the microbiome to the human metabolome and to study their interactions.^51–53^ Associations between nutrition, microbiota, and immune system are being actively studied as contributors to chronic metabolic diseases.^54^

The application of the multi-omics approach has been shown to be of great interest to better characterize the complexity of phenotypes in human cohorts, but its translation to the clinical setting remains to be developed. Technical advances in biomarkers and personal monitoring devices open the door to translate the concept to utility and increase the completeness of the human system. Integrating communication tools and the exposome as a full part of systems in medicine is now under development, as are analytics that can make full use of the complexity of multidimensional omics data (see the section about “Embracing the challenge of data integration and validation”).

### Big data availability and information systems

The emergence of powerful approaches allowed large datasets to be produced and analyzed, in the perspective of developing decision-making tools for health management. One of the challenges is the security of personal and private health data.^55^ Moreover, the generation of high volumes of big omics data, combined with the health care provider's high rate of data generation (also known as data velocity) constitute a critical challenge for supporting research and practical implementation of system medicine and tools. Securing personal and private health data is an additional crucial challenge for managing systems. Therefore, the future development of systems medicine requires advanced informatics tools for merging the different kinds of data to be shared among different communities.

### Findable, applicable, interoperable, and reusable, privacy and federated machine learning

Big data also harbors risks to the safety of sensitive clinical data, in particular, when such data need to be copied to clouds to provide software for learning statistical models with the required large-volume, high-quality data. The barrier for secure health data exchange over the internet is perceived to be insurmountable, thus posing a massive bottleneck hampering big data and prohibiting progress in computational systems medicine. Therefore, it makes the development of medical artificial intelligence (AI) tools for prognosis, response prediction, or treatment optimization *de facto* impossible, as sharing and cloud-based storage of health data is ethically problematic and often legally prohibited.

Modern omics technologies have paved the way for large-scale quantitative profiling of all kinds of biomolecules (genome, mRNAs, proteins, small molecules). With such data for many patients, we can build computational models that can predict medically relevant features (biomarkers). The PAM50 gene signature and the MammaPrint panel are such biomarker models,^56,57^ helping clinicians to determine whether a breast cancer patient will benefit from chemotherapy, and from what kind of chemotherapy. However, recent results raise concerns regarding their predictive clinical value.^58,59^ The major problem is the selection of biomarkers due to the small number of samples compared with a very high number of features. The Cancer Genome Atlas^60^ and the International Cancer Genome Consortium data portal^61^ are, by far, the most comprehensive repositories for clinical cancer omics data worldwide. For breast cancer, gene expression data for <2000 patients are available. These few thousand samples, however, stand against more than 20,000 genes that AI may combine to predict the outcome. Even when following best practices in machine learning (ML), the consequence is model overfitting and a significantly reduced impact of such kinds of AI-based medical diagnostics tools.

Big Data is clearly in its infancy, even in oncology (the most advanced research area of precision and systems medicine). At the same time, one in eight women (ca. 12%) will develop breast cancer. In the European Union (EU), there are more than 350,000 new cases per year.^62^ How come that we need to train ultra-high-dimensional AI models with >20,000 features (genes) on <2000 breast cancer samples, whereas in the EU alone >350,000 new cases occur every year that are often investigated by using gene expression (PAM50, MammaPrint)? Even if only 20% of them were analyzed computationally, and even if only 50% of the samples would be of sufficient quality to be used for AI learning, over the past 5 years we could have accumulated >300,000 samples—in contrast to the 2000 samples mentioned earlier, exemplifying how far we are away from Big Data analytics in precision and systems medicine.

Legal and ethical considerations dictate these circumstances. Patient data may not be shared, in particular neither the molecular data nor the electronic health records, and most certainly not over the internet. In contrast, conventional AI tools require access to all data locally for training, resulting in the need to aggregate available data in a centralized cloud repository. But data protection legislation usually prohibits depositing sensitive medical patient data in central storage outside the hospital, with massive consequences. For example, the EU's laudable attention to privacy and respective national legislation is further feeding this problem and creating contradicting requirements: The General Data Protection Regulation and its national implementations, as well as the criminal laws on confidential medical communication and the restrictions in terms of data “ownership” prohibit the exchange of sensitive patient data,^63^ whereas simultaneously the findable, applicable, interoperable, and reusable (FAIR) principles are enforced, for example, in the H2020 programme, where projects are required to make research data publicly available.^64^

One potential way out of this dilemma is federated ML. However, many challenges are to be overcome until client-sided ML becomes ubiquitous^65^; however, recent experiments on deep learning demonstrated that it can be made practical and that there are many intriguing opportunities.^66^ Europe has dedicated research projects to this task, for example, FeatureCloud, but no applications for federated systems medicine (e.g., for federated network enrichment, federated composite biomarker extraction, or federated mechanotyping) exist yet; however, it would be necessary to make systems medicine big-data-ready.

From a Health care Services perspective, data availability is a sensitive matter. For example, in the United Kingdom, the National Health Services is financed by the government but health care customers' data are not centralized. In contrast, in some countries, such as Denmark^67,68^ and Israel although the health care system is also financed and regulated by the government, health care customers' data are centralized. In Israel, these data are centralized by the Healthcare Management Organizations (HMO) whereas some of the data reside at hospitals and the Health care ministry. The data available to the HMO include, among others, socio-demographic data, and information on biological tests, clinical examinations, pharmacological treatments, and communication channels. These data have been continuously collected and stored at the health care customer level for the past 25 years.^69^ In Israel, the health care data *de facto* fit the FAIR standards.^70,71^

### Standardization

Large datasets are often generated at great cost, consuming significant time and resources. Critical to realizing their full value is that we can quickly and easily deploy a diverse and well-developed set of software tools for analysis. This is best achieved when the datasets are made available following a common set of data standards used by a wide range of software tools. Conversely, the incentive for developing software tools is invariably stronger when a rich landscape of suitable data already exists that is easily accessible *via* data standards. Hence, data standards are vital to realizing the potential in large datasets. The adoption of Binary Alignment Map and Variant Call Format file formats, for example, has underpinned the explosive growth in the availability of genome data and the software tools for analysis. As we move into the era of systems medicine, data standards will be central to maximizing the value we derive from systems-level datasets.

Larger and more complex datasets require more sophisticated analysis and, as analysis grows in sophistication, it becomes increasingly challenging to reproduce. This is in part due to the network of software dependencies associated with the analytical tools and in part due to the array of design choices that form part of the analytical workflow. Hence, we not only need standards to optimize data availability but also need standards that make analyses reproducible and verifiable.

Among the key current standards that will support the growth of systems medicine are the Systems Biology Graphical Notation (SBGN), a set of symbols and usage rules that have been developed through open community action, as tools for mapping out the network of molecular interactions between genes, proteins, and small molecules.^72^ Although many mapping systems exist, SBGN's strength lies in its lack of ambiguity and its machine-parsable structure, which means that maps can be translated straight to mathematical models. Three different flavors of SBGN have been created with the Process Diagram, providing the most details and the highest level of parsability whereas the activity Flow and Entity Relationship provide greater levels of abstraction and lower levels of parsability.^*^

Such models typically require great effort to develop^73,74^ and to facilitate their reuse, expansion, and refinement, they can be made available by using the SBGN-ML file format that captures the structure of the maps and encodes the biological meaning of the symbols.^75^ It is based on coded use of plain text and therefore it can be edited not only in SBGN-ML compliant software but also manually in any text editor. The mathematical model that facilitates simulation of the pathways can be encoded and disseminated by using the Systems Biology Markup Language (SBML). Similar to SBGN-ML, it makes use of a coded plain text file format that can be edited either by SBML compliant software or manually in any text editor.^76^ However, SBML captures the system of Ordinary Differential Equations that describe the kinetics of all the interactions between genes, proteins, and small molecules in a machine-parsable form along with their structure and meaning.^†^

Ensuring the reproducibility of simulations and computational experiments requires the adoption of the standards for the maps and models mentioned earlier as well as a further set of standards to describe how they were used. In particular, the minimum information about a simulation experiment (MIASE) standard requires users to (i) specify and make available the exact model used, (ii) specify exactly how the model is simulated, and (iii) specify how the outputs are calculated from the model.^77^

The MIASE standard is descriptive and, therefore, exposed to the subjectivity of the author. A more comprehensive approach is to capture (i) all the codes that have been used to analyze the maps/models, (ii) all the outputs from the code, and (iii) a copy of the software used to run the code all in one place, so that this collection can be disseminated and other users can rerun the analysis and edit to experiment with the map or model. This is now possible with the advent of interactive scripting. Among the most prominent examples is the Jupyter Notebook in which authors can create word processor standard documents with code and code outputs embedded in the document along with a programming environment that enables the code to be executed.^78^ Jupyter Notebooks originally supported the Python, R, Haskell, and Ruby programming languages, but the list of supported languages has since grown considerably. MATLAB supports similar scripting with its Live scripts, though they only support MATLAB's scripting language.

The ultimate approach to disseminating maps, models and how they have been analyzed is to take a snapshot of the computer on which they have been run and to transmit this snapshot. The snapshot contains a copy of the code and software needed to run the code, all the relevant files from the hard disk of the computer, and all the relevant parts of the operating system needed to run the software. The Docker platform achieves this by creating minimal virtual machines called containers that hold everything needed to run the analysis.^79^ These container files can be distributed to other users who can run them on their computer by using the Docker engine. Because the containers hold everything needed to run the analysis, other users do not need any compatible software beyond the engine. This not only reduces the challenge of rerunning the analysis but also eliminates the challenges of cross-compatibility between platforms and operating systems.

Therefore, one of the most important points in systems medicine is to develop conceptual models for their integration.^80^ Once a computational model of the disease pathophysiology is available, a systems medicine model allows the setting of experiments that would not otherwise be possible for logistical or ethical reasons, especially around the iterative development and refinement of new mono- or multidrug therapies.

### Embracing the challenge of data integration and validation

Several discussion articles and reviews exist on omics data integration, from the perspective of model organisms,^81,82^ including microbes^82^ and bacteria,^83^ or from the perspective of humans,^84–86^ and host–microbe interactions.^87^ The available tools and methods of integrative omics analytics are not sufficient, and they even fail to successfully integrate, let alone analyze, different levels and sources of omics data. Important lessons can be learned from smaller-scaled analysis efforts. For instance, only adding one level of complexity to GWAS, namely multiple marker interaction analysis, has been a sobering lesson.^88^ It has pointed toward problems that need to be tackled in omics integration efforts as well, as they are expected to be elevated when dealing with multiple non-independent, possibly interacting, dimensions. These problems include significance assessment, heterogeneity modeling in meta-analysis to increase power, replication, validation, and replication^88,89^ and are widely applicable to systems medicine modeling in general.

Traditionally, integrative analysis techniques have focused on combining evidence derived from real data combined with public database knowledge.^90,91^ The field then moved on, from exploiting the combination of private and publicly available knowledge to accelerate drug discovery,^92^ to combining multidimensional views in, for instance, gene mapping.^93^ Method developers are only gradually pacing up with the vast amount of heterogeneous data sources that become available and with introducing the necessary complexities into the models.^94,95^ With omics data increasingly being collected on the same set of individuals, it becomes theoretically possible to connect different layers of cellular or molecular information (for instance in causal models^96^), while combining analytics to available expert knowledge.

Integrative tools for Big Data ideally combine kernel theory (to bring in notions of nonlinearity), components theory (to reduce dimensionality), and graph theory (to handle dependencies and interactions in systems). From a practical point of view, it remains essential to understand the minimum requirements that each analytic tool for omics integration should have for it to be able to distinguish “noise” from “signal” and to compensate for the intrinsic power deficits resulting from having relatively small numbers of individuals with huge numbers of omics measurements. The metabolomics community has recently published viewpoints and recommendations for the development of multi-omics integration in the context of systems biology.^97^ “Special Issues” on omics integration (e.g., Genin and Devoto)^94^ highlight remaining challenges, including the integration of dependent and independent omics datasets in meta-analyses,^98^ and the integration of omics with non-omics data.^99^

**FIG. 2.**
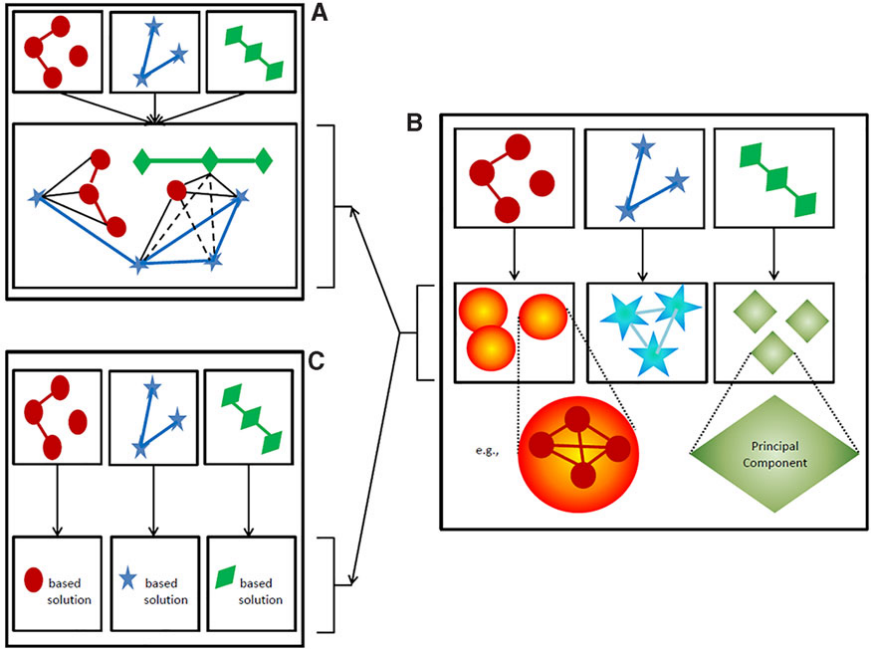
Traditional choices to handle different omics data sources before deriving an integrated solution. Different colors and symbols represent different data origins. **(A)** Data fusion, which allows accounting for structure between omics data. Evidence for such structural relationships may be derived from biological knowledge or analytically (full lines), or it may be deduced from the latter (dashed lines). **(B)** Changing the representation of each data source. This may be based on principles of dimensionality reduction or the identification of communities (cf. corresponding data corresponding symbols with gradient fill). **(C)** Obtaining a data-specific solution, hereby ignoring detailed inter-relationships between data sources as part of obtaining an integrative solution. Once data are represented as in **(B)**, cross-data source relationships may be accounted for **(A)** or specific within-data source solutions may be targeted first **(C)**, before obtaining an integrative solution. This is indicated by the arrows connecting panel B with, respectively **(A**, **C)**.

Once omics data have been curated, Hamid et al. have identified three general roads to travel.^84^ Either the data are fused before modeling (Fig. 2A) or the representation of each omics data sources is altered to make it more digestible before deriving an integrative solution to the problem of interest (Fig. 2B), or each omics dataset is modeled separately and results are integrated (Fig. 2C). Although these stages of integration are often discussed in the framework of association analyses, they also apply to prediction and profiling (pattern recognition) contexts. Notably, most of the novel analytic approaches to integrate multiple omics dimensions do not concern analysis on fused data (Fig. 2A).

The main reason is that such analyses imply quite many information technology–infrastructure and computational challenges as well as analytic challenges in that any model is believed to be a too severe over-simplification of the rich information that the inter-related data potentially entails. There are different omics levels of informativity and errors to account for, as well as for different measurement types and patterns of missingness. Regardless of this, fusing data before analysis seem to be the only natural way to fully account for non-independence between omics data records and the analysis of data according to the spirits of systems biology. Does this mean that omics integrative analysis will be hopeless for many years to come? We do not think so…let us be creative!

### Out-of-the-box thinking

**FIG. 3.**
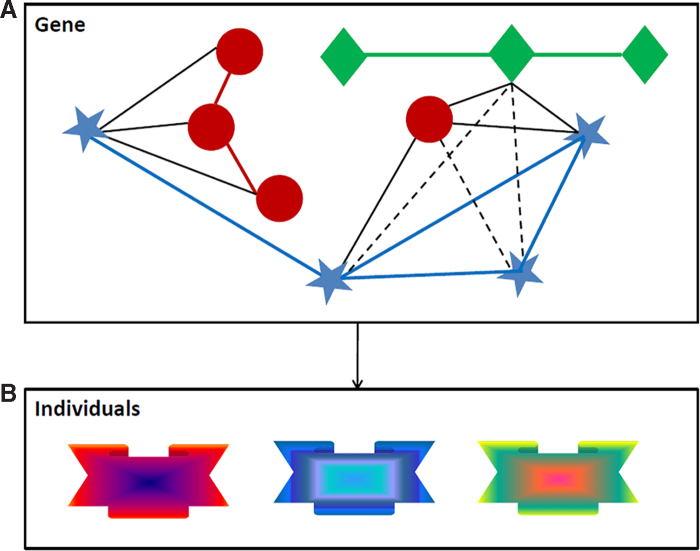
Fully acknowledging inter-relationships between omics data on reduced genomic sets when deriving integrative solutions. **(A)** Per meaningful genomic concept, such as a gene, create a network of inter-relationships between omics elements “mapped” to that concept. **(B)** Represents the concept-based integrated data by using kernel-based principal components, where the kernel is chosen in such a way that the structure of the data is optimally captured. This leads to a new integrated concept-related signature for each individual in the sample. Each concept, therefore, gives rise to a new variable. The combined set of concepts (new variables) is submitted to subsequent analyses to obtain an integrated solution to the problem of interest.

One of the ways to overcome the computational burden and analytic complexities described earlier is to re-define the boundaries of the system we wish to elucidate. Taking the example of gene mapping, we can take a “gene” as a mini-system (Fig. 3) and combine principles of data fusion (Fig. 2A) with ideas to change omics data representation (Fig. 2B). In particular, we first capture the relationship between a meaningful set of omics features (Fig. 3A) and then change the representation of that set (Fig. 3B) while converting it into a single aggregated feature (i.e., a multidimensional module). Structure within each set can be modeled *via* prior knowledge or analytically on the observed data, such as *via* partial least square-based path modeling that offers more possibilities than classic principal components analysis.^100^ Such a strategy can be applied to any meaningful “unit of analysis” with characterizing features that can be represented as a network. From our perspective, the most promising strategies within an omics integration process are based on components-based association modeling,^101^ diffusion kernels on graphs for prediction,^102,103^ and similarity network fusion profiling.^104,105^

### Validation

One critical step of modeling approaches is validation, as fitting a model to data does not prove that it will accurately capture the clinical outcome. In particular, the high dimensionality of datasets is a major challenge in data analysis, especially for model reliability, as it is prone to overfitting. Therefore, there is a special need to develop dedicated protocols for validation of integrative (systemic) analyses. This effort requires tools that enable simulating realistic and sufficiently complex data. Consequently, simulation-oriented approaches have been increasingly applied over the past 5 years. Different alternatives exist in terms of validation strategy:

1. *In silico* data generation: As an example, a multi-omics data simulator for complex disease studies was developed and applied to evaluate multi-omics data analysis methods for disease classification.^106^ Another tool, iOmicsPASS, allowing network-based integration of multi-omics data for predictive subnetwork discovery was recently published.^107^
2. Validation protocols and the interpretation of validation studies: In contrast to replication, validation in other samples does not require sampling from the same populations as the discovery study. This poses particular challenges toward interpreting the results from a validation study due to sample heterogeneity. Especially when thousands of features from heterogeneous data types are being collected, the problem of heterogeneity between individuals—assessed *via* the collected data—may become more pronounced.
3. Preclinical validation suffers from the limited interpretability of *in vitro* cellular or *in vivo* animal models. Currently, we do not know for most diseases the underlying mechanism, which makes it close to impossible to decide whether the animal model that mimics a human disease symptom is due to the same mechanism. Once we know the human mechanism, there will be almost no need for an animal experiment; alas a drug repurposing study requires this, for example, for regulatory reasons.
4. Clinical trials have to be viewed as ultimate validation and with the shift from imprecise symptom-based disease definitions and symptom-based therapies, we will be able to design much smaller highly precise mechanism-based interventions with small NNT, up to n-1-trials.

Defining the network and systems medicine framework is now allowing us to disclose some current and future clinical applications. In the next section, we elaborate on how systems medicine is being implemented on the field and in the real world.

## Clinical Applications (on the Horizon)

Systems medicine is starting to be greatly used not only in the context of cancer but also in pharmacology. It has opened the door to advanced personalized medicine in these areas, improving the clinical approaches.

### Cancer pathways and personalized therapy

Recent personalized therapeutic approaches in oncology target multiple pathways within a mechanistically defined cancer type by combining several drugs with the aim of curing or at least significantly improving survival and quality of life beyond current symptomatic or cytotoxic approaches. In this context, the increasing availability of pathway knowledge that is relevant for human systems modeling, for instance from databases including Signor^108^ and Reactome,^109^ provides quite extensive information for building cellular signaling networks that allow the analysis of cancer cell function.

**FIG. 4.**
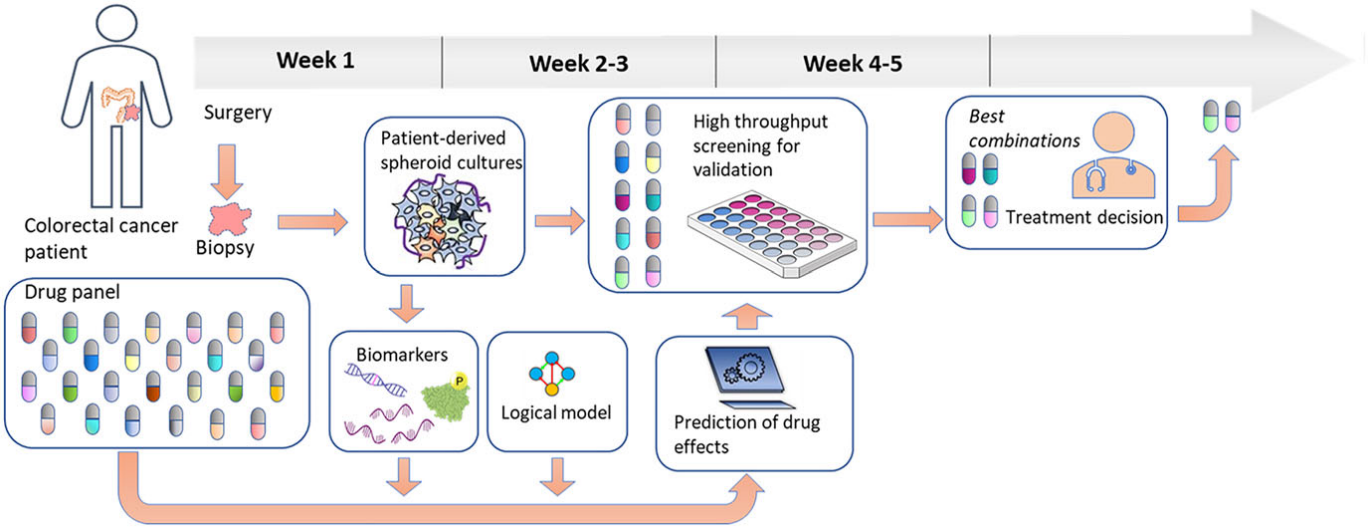
The proposed implementation of logical model predictions and patient-derived spheroid testing of drug therapies. An individual patient's tumor material (top row) is used to produce spheroid cultures for small-scale drug combination screening. In parallel, biomarkers are produced from these spheroids (bottom row) and used to configure a generic logical model so that it optimally represents the tumor of the patient. This model is used for a large-scale *in silico* screening of the complete available drug combination space, resulting in a limited set of potential synergistic drugs that are tested in the spheroids. This whole procedure can be completed in a couple of weeks. Validated drug combinations can be considered by a clinician for therapy decisions.

The conversion of such a cell fate decision Prior Knowledge Network to a Boolean model is in practice a relatively trivial task, starting with the use of the causal interaction information to generate the logical rules that mathematically define the interactions of the network as a whole. An accurately designed logical model of a cell will follow these rules to arrive at a stable state in which the activities of the model components will quite accurately represent the activities of their biological counterparts in the cell that is represented. Logical models built using Signor data, complemented with some additional *ad hoc* literature curation, have allowed, for instance, the assembly of several versions of a colorectal cancer model that have quite significant predictive power in assessing the effect of combinations of targeted drugs^109,110,‡^ on cellular states, and they can be used to identify potential synergistic drugs that together are more effective for inhibiting cell proliferation than separately. The procedure to do this is as follows: A general logical model is configured to represent a specific cancer cell line using baseline biomarker data that inform the logical model about the activity states of Boolean network nodes (Active=1, Non-Active=0), and the resulting cell line-specific model can be used to filter out *in silico* the combinations that are least likely to display synergy. The remaining potential synergistic drug pairs can subsequently be tested in cancer cell line cultures to validate the synergy predictions.^111^ From this proven system, the next challenge is to implement it in a clinical setting, and to develop patient-specific logical models by using biomarker data from tumor biopsies obtained from a cancer patient, use these to select potential synergistic drug pairs, and test these on *in vitro* cultured spheroids or organoids derived from the same tumor material (Fig. 4, see also refs.^112,113^). The timeline needed to perform such an analysis would be a matter of weeks, during which the patient would receive standard postoperative chemotherapy.^108,109^

### Personalized drug therapy

#### Drug dosing

Precision medicine will also be oriented to personalized drug dosing to improve their efficacy and safety. In this roadmap, two important problems need to be addressed. On one hand, it is necessary to design personalized drugs for each disease and patient. New opportunities are arising with the arrival of technologies that allow the printing of three-dimensional (3D) drugs. Thus, new improvements such as personalized dosages, chewable pills, multi-active pills, and fast-dissolving tablets have been proposed. The second problem in the improvement of treatment efficiency is related to the administration of drugs to the patient. In general, very low differentiation in dosage is done for the same disease in similar patients. However, even individuals who share similar characteristics have different responses to drug administration (inter-patient variability). Also, for the same patient, the response to drug administration can vary with time (intra-patient variability). This can occur due to changes in the patient's condition during the treatment.

Systems medicine arises as an approach that can help in the personalization of drug dosing. One of the key ideas in the development of personalized drug dosing mechanisms is the concept of closed-loop or feedback control. This concept plays an important role in both engineering applications and natural systems. The main idea behind it is the observation of the output variable (variable of interest) to decide how to modify the input that is applied, to change the value of the output variable.

One of the most relevant medicine areas where feedback control systems are being applied is anesthesiology. Three main variables are involved in general anesthesia: hypnosis, analgesia, and neuromuscular blockade. For each of these variables, a different drug is applied. The anesthesiologist needs to estimate the correct dose for each of these variables. In traditional clinical practice, drug dosing is according to patient characteristics (body mass index, age, gender, and height). During the process, the anesthesiologist corrects the drug dose according to the patient's response to drugs.

If more accurate and safer drug dosing is desired, closed-loop control appears as the best option to be considered. The design of advanced control systems in anesthesiology involves methodologies included in the systems medicine approach. In particular, three main challenges need to be addressed:

1. Effect assessment: The first step toward personalizing drug infusion is the availability of an index that correlates well with the variable of interest. For general anesthesia, different measures have been proposed to measure the unconscious level of the patient:^114,115^ Bispectral index (Medtronic), Spectral Entropy (Datex-Ohmeda), Narcotrend index (Monitortechnik), Patient State Index (Masimo), or Auditory Evoked Potentials Index (Danmeter). For neuromuscular blockade monitoring, there are also reliable techniques, and most of them are based on Train-of-four stimulation.^116^ However, one of the current challenging issues is the proposal of a reliable index to assess analgesia level in patients. This is a much-complicated problem due to the complexity of the involved mechanisms and the disturbances affecting the process. Current monitoring devices for analgesia focus only on one or two variables (electroencephalogram signal, electrocardiogram signal, respiratory frequency, pupil diameter, mean arterial pressure, photoplethysmographic signal, etc.) to generate a pain measurement for the patient.^116,117^
  It seems that a more general focus should be considered at this point. Thus, systems medicine proposes new approaches based on the development of new indexes for drug effect assessment based on the integration of multiple sources of information. This could lead to more reliable indexes that can be used to implement efficient and safe feedback control systems.
2. Modeling of patient response: The improvement in the design and personalized titration for drugs greatly depends on the availability of reliable models. The aim is to be able to predict patient response and use this information to design personalized drugs and to administer them. Different methodologies can be used to model patient response. Thus, the main methods for this are physiological models (built on the basis of physiology, anatomy, and biochemistry of the body), compartmental models (based on the assumption that the body can be represented as a set of interconnected compartments),^118^ and black box models (representations of the functional relationships between system inputs and system outputs). Compartmental models are much simpler than physiological ones and have been intensively used in practice. In the past, many studies were conducted by using black box models, based mainly on neural networks, fuzzy logic, evolutionary computation, and ML^119,120^
3. Drug infusion control systems: The third great challenge for personalized drug dosing is the design of efficient controllers to decide the correct drug dose that the patient needs. In the field of general anesthesia, three main possibilities can be found. The first option is the signal-based controllers. These strategies are mostly based on proportional integral derivative controllers. The algorithm decides the drug dose according to the measured errors observed. The performance of these methods is satisfactory, although they have the inherent limitation of using only information of the history of the patient.
 Alternatively, model-based controllers predict the response of the patient (using any of the methods described earlier) and compute the solution that optimizes the response of the patient.^121^ These controllers, also known as predictive controllers, greatly depend on the reliability of the prediction model. A third option is intelligent controllers that include all those methods based on AI techniques. It is common to find applications using fuzzy logic control that are based on heuristic rules.^121,122^ These methods allow a direct translation of the expertise of the anesthesiologist to the computer. Besides, applications based on neural networks can be found.^123^ The ML techniques are also being used for the design of computer-aided decision systems for the anesthesiologists.^124^

Current research in anesthesia control systems is focused on three main issues. The first is the design of a control system with robust capabilities to reject disturbances occurring in the operating room. This is of great importance, as the patient is affected by many stimuli that change during the surgery. The second important issue is the study of the interaction effect of different drugs. If an optimal drug dose is desired, it is necessary to study the patient's entire system and his/her response to the different drugs that are being administered. A third important problem during surgery is related to the changes in the response to drug infusion between different patients (interpatient variability). This means that the controller must be able to offer a satisfactory response regardless of the patient profile. The problem also occurs for a given patient during the surgery, as his/her response to drug infusion changes with time (intra-patient variability). The solution is the inclusion of adaptive systems in the closed-loop system. The controller should be able to adapt to the observed patient response. The complexity of this problem makes it necessary to use systems medicine approaches to help not only in the description and prediction of patient responses but also regarding the design of robust and efficient controllers.

This discussion about key issues in anesthesiology and the need for new approaches based on systems medicine opens up new perspectives for future research. It is important to note that most of the concepts explained for general anesthesia drug infusion can be extended to any other discipline in medicine.

#### Two-dimensional- and 3D drug printing

Network and systems medicine will enable not only individual and genetic diagnoses but also precisely designed therapies—currently mainly pharmacotherapy. Individual pharmacotherapy is not entirely new, for example, it has always been individual in infusion therapy. Patients suffering from a tumor, for example, receive a therapy tailored to their needs, in which the strength or dose is precisely matched to the patient. Type 1 diabetics inject exactly as much insulin as they need in the respective situation. In drug therapy with tablets or capsules, individualization stops. Patients receive the active ingredient or combinations of active ingredients available on the market, each in a standardized strength or dose. These active strengths and doses are determined in phase I–III studies that do not represent the collective in which they will later be used. The one-size-fits-all philosophy dominates therapy with oral drugs and thus prevents possible individual pharmacotherapy. This is particularly problematic in patients with impaired organ function (e.g., kidneys) or with active substances with a narrow therapeutic window. In addition, various drugs are metabolized hepatically. This varies greatly between individuals and to a clinically relevant extent (e.g., Clopidogrel, Tamoxifen).

Even the introduction and elimination of therapies is currently only sub-optimally possible, with the help of digital printing technology, it is now possible to print the active substances required by a patient in exactly the strength or dose and in the needed combination. Patients, in particular those who have to take several tablets, will benefit because they will no longer have to take several tablets but only one in the future, which will contain all the active ingredients in the corresponding strength and release kinetics. Three-dimensional printing technology^125^ uses digitally controlled devices for formulating active pharmaceutical ingredient (API) and excipients in a layer-by-layer pattern for developing a suitable personalized drug delivery system as per the need of the patient. It includes various techniques such as inkjet printing (two-dimensional printing), fused deposition modeling (3D printing), which can further be classified into a continuous inkjet system and drop-on demand.

To formulate such dosage forms, scientists have used various polymers to enhance their acceptance as well as therapeutic efficacy. Polymers such as polyvinyl alcohol, poly (lactic acid), poly (caprolactone) etc. can be used during manufacturing. A varying number of dosage forms can be produced by using 3D printing technology, including immediate-release tablets, pulsatile release tablets, transdermal dosage forms etc. Thus, it is foreseeable that in a few years, patients will receive not only highly individualized diagnoses but also personalized, precision therapies, which, by combining APIs into one printed tablet, will also increase compliance. With the help of digital printing technology, it will be possible to perform compounding with oral drug therapy. Ultimately, this technology will lead to higher efficiency and effectiveness and, consequently, significantly reduce overall therapy costs.

#### Drug repurposing

To have an impact and induce the necessary changes in our approach to medicine, network and systems medicine needs to provide clinical evidence. If this would involve new targets and depend on drug discovery and drug development, the proof-of-concept for network and systems medicine would take at least another 15–20 years. This gap can, however, be overcome by drug repurposing, that is, the reuse of a registered drug for a new indication. By repurposing a registered (set of) drug(s) for a new indication, nearly the complete lead optimization and most or all of the clinical phase I is eliminated. Ideally, immediate phase II clinical trialing is ethically possible and medically justified, provided solid preclinical evidence on the target and drug can be provided. Compared with having to start from lead discovery, the net gain in time is at least 9 years on average. Depending on the indication (acute or chronic) and resulting trial length, the gain may be even more.

This process is not new but has so far rather been serendipitous and projects such as the EU-funded Horizon 2020 project REPO-TRIAL (repo-trial.eu) takes this to another level and makes it more predictable and precise. REPO-TRIAL, a 5-year project, focuses on indications that allow short-duration trials, either because the patient-relevant outcomes can be observed within days or weeks (stroke, myocardial infarction, resistant hypertension) or because predictive biomarkers are available (diastolic heart failure, gout). Ultra-short or short trials are increasingly common and acceptable from a regulatory point of view, in particular for the most likely initial phase II, safety phase, with efficacy parameters rather being secondary outcomes. In REPO-TRIAL, a cluster of comorbid disease phenotypes has been associated with dysregulated reactive oxygen and cyclic GMP signaling. Patients are stratified based on biomarkers indicating this dysregulation and then treated with repurposed registered drugs that target these signaling pathways.

The first trials on stroke (REPO-STROKE) and heart failure with preserved ejection fraction (REPO-HFPEF) are expected to be finalized in 2021 and 2022, respectively. With 2538 approved small-molecule drugs (Drugbank), the likelihood is high that for any given causal network at least one drug would be available. Indeed, this is the case for most targets. A fascinating recent observation, based on the PISCES dataset, is that registered drugs bind with high affinity to conserved binding pockets of, on average, 39 proteins.^126,127^ Thus, small-molecule drugs are highly promiscuous and, in all likelihood, can be repurposed from one to many other target proteins with similar binding pockets. Repurposing registered drugs with known safety profiles may be so powerful that they may rapidly address therapeutic needs in many other causal disease pathways and thus outcompete classical drug discovery. Moreover, drug repurposing has occurred earlier, but mostly in a serendipitous manner; with network medicine this will become highly predictable, pathway by pathway.

## Enlarging Network and Systems Medicine Applications

### Improving patient engagement and treatment adherence

Network and systems medicine applications are further enlarged by improving patient engagement and treating adherence. Day-to-day health care services are not based on genotyping but rather on phenotyping. How to treat a patient is generally based on a physical examination and understanding a patient's behavior. Accordingly, improving patient engagement and treatment adherence strongly relates to the concept of the exposome, which, among others, deals with the complexity of patient–caregiver interactions and other environmental factors such as the sociological and economic factors.

In recent years, the number of channels allowing health care customers and practitioners (a.k.a. providers) to communicate has grown dramatically. These channels are one dimension of the exposome, allowing a measurement of the strength of the interactions between the health care systems actors. Historically, patients and providers used face-to-face meetings as a standard means of communication. When phones were added to the health care organization arsenal, they were used for scheduling appointments or asking for services such as prescription renewals or medical recommendations.^128–137^

Since the beginning of 2010, the advent of the Internet, and the popularization of smartphones and social media, the rules of communication between health care customers and health care providers have profoundly changed.^138,139^ This digital revolution is also allowing the health care system to integrate new tools supporting teleconsultation and tele-diagnostic systems, and to continuously develop and integrate innovating tools for both patients and health care providers.^140,141^ The main purpose of a large number of communication channels available today is to provide new ways to search, get, and share health-related information and knowledge. Nevertheless, the level by which health care customers and practitioners used these channels depends on numerous environmental factors such as economics, culture, and regulations. The interactions between health care customers and practitioners must, therefore, be tracked and integrated as part of the system medicine data, as exposed-generated data, to provide an overall understanding of the patient so that the treatment and the educational and therapeutic messages are delivered to each patient in the most suitable way.^142^ Consider the following real-life example: HMOs record medical data and their interactions with their insured health care customers.

One way by which patients' engagement and treatment adherence can be improved is based on the identification of subpopulations of patients by considering their communication usages and then characterizing each one with socio-demographic and bio-clinical data for improving treatment effectiveness and treatment adherence. This approach has been implemented, in 2015, on 309,460 patients with diabetes and 7 dominant profiles have been discovered and characterized to help health care decision makers to improve follow-up policies and tools. Personalized services focusing on patients' needs and preferences were implemented based on this analysis.^140,141^

Altogether, to increase the frequency of successful translational stories, the research enterprise needs to re-design research studies by considering the complexity and variability of human physiology, and by collecting high-dimensional datasets that will allow researchers to identify confounding variables and to stratify populations at early phases of biomarker discovery. As the “omics” term is expanding to wider systems, all of these have to be interrelated.

One of the remaining challenges of health care systems is patient accessibility. One way to improve this is to find the most suitable communication channel(s) to interact with a patient based on his/her profile, which combines socio-demographics, clinical, biological, and therapeutics data over time. This approach induces, at least for part of the population, proactive behavior and engagement in follow-up and treatment when relevant.^140,141^ The HMOs around the world are developing digital services, such as online counseling services, which integrate videocalls to physicians when the clinics are closed. This kind of consultation is based on the overall patient's data shared over the electronic medical record, thus allowing any health care practitioner to have a clear view of the patient anamnesis and therefore delivering low-biased recommendations and treatment. Sharing data is an essential part of developing and delivering personalized medicine. As an example, when searching for patterns of interactions of patients with diabetes, an Israeli HMO allowed pointing out the need to tune its communication tools and messages to patients, more particularly to those with special needs, such as elderly people, immigrants, and minorities, who are not fluent in the local language, and those with low socioeconomic status. Matching a communication tool and message to the patient will improve the patient's accessibility to HMO services, generate a better patient engagement and responsiveness to treatment, and improve the quality of treatment and treatment experience within existing budgetary constraints. Particularly, for patients with diabetes communication is a key dimension of systems medicine, which will provide an opportunity, for example, to collect more patient-reported outcome measures^143^ for some basic follow-up measurements such as glycemia values, weight (for computing body mass index), and smoking status.

### Training

There is a consensus that systems medicine-specific training is a need, recognized by trainers, students, but also by the authorities. A major challenge of today's medicine is the ability to integrate the technological revolution, expansion of data collection that comes in multiple formats and is stored in different computers at different clinical sites, into the coordinated everyday clinical practice. Many of us believe that one, or even two generations of new medical doctors (MDs) and researchers might be needed for this to be achieved. We also believe that a society must educate their new generations on the data and technology revolution in medicine. The younger generations are already sensitized to comprehend and adapt to these changes due to their experience dealing with new technologies (smartphones and other gadgets, social media, etc.)^144^

Although the medical community is becoming increasingly aware of these educational needs, the how (and when) to introduce these new subjects is not so obvious. One view is to apply systems biology approaches and tools to biomedical problems, and to start educating biomedical students in an interdisciplinary manner as early as possible. In addition, these educational efforts have to take into account ethical concerns as well as economic circumstances and specific aspects of the different health care systems. Therefore, a joint effort of all (bio-)medical education and health care delivery stakeholders is key in this process. It seems evident that systems medicine training of future physicians cannot include a deep education in programming, mathematical modeling, or computational sciences—given the wealth of medical information that has to be tackled in the course of medical studies. Instead, medical students need to learn the skills of using professional software solutions that have been developed by specialists in an interdisciplinary manner, together with practicing doctors. To state it simply, a car driver does not need to know and understand in detail how the engine of a modern car works but has to know how to drive the car. Similarly, future MDs have to know how to apply systems medicine solutions that have been developed by specialists in their daily medical routine.

#### Undergraduate education

Despite the recognized need to change and adapt the education programs of (bio)medicine studies, there is no agreement on the best practices and ways to achieve this goal.

The reason lies in the generally fragmented approach in the European higher education system, where even within a single country, universities teach similar subjects by different principles and keep the decision autonomy. Universities are independent in offering novel courses; accreditation for these is requested in countries that follow the Bologna process. Some steps toward the implementation of a systems medicine education have already been tested within the FP7 CASyM (Coordinated Actions Systems Medicine) and later within EASyM (European Association for Systems Medicine). Similarly, the International Network and Systems Medicine Association provides such resources at an international level and is a direct spin-off of the Collaboration on Science and Technology action OpenMultiMed.

Several medical schools in Europe teach subjects that are relevant to systems medicine. What is missing is the combination of relevant subjects into modules that would receive the formal name of “systems medicine.” If such modules are provided mostly into elective courses, we should ensure that students receive the proper information regarding the systems medicine subjects. For example, at the Faculty of Medicine, University of Ljubljana, systems medicine topics are currently covered within computational and practical/research elective courses in (bio)informatics, mathematics and computer-supported approaches, and e-learning; whereas in senior years, interdisciplinary courses are given in functional genomics and pharmacogenetics. At Maastricht University, the Netherlands, medical students can choose a Network and Systems Medicine elective.

#### Doctoral education

Similar to the undergraduate situation, doctoral education is also dispersed in Europe. Three possibilities appear feasible in the future:

1. Introducing (accredited or non-accredited) systems medicine concepts or subjects into the existing biomedical doctoral programs.
2. Introducing novel interdisciplinary systems medicine research training networks for doctoral students within the ITV Marie Curie or similar programs.
3. Establishing a formal systems medicine doctoral program at individual universities. This option has not yet been tested in Europe but is active in the United States. The Georgetown University MS degree in Systems Medicine is designed for students interested in bringing systems medicine into biomedical science and clinical practice and setting the stage for bridging research and clinical care. The MD/MS dual program is designed for students already accepted into medical school, and who will take an additional year beyond the four required for the traditional MD to complete the MS. Students will be accepted into the program after completion of their second year in medical school.
  The program educates physicians to understand and apply new approaches to diagnose, prevent, or delay disease manifestation and improve clinical outcomes for patients. The MS and MD/MS Dual Degree programs in Systems Medicine teach students to use cutting-edge technology to train the next generation of physicians and biomedical scientists. Students learn a new language, which is the application of omics technology and Big Data to patient care. In addition to credits gained by courses, students carry out a semester-long Capstone internship where they gain hands-on work experience in renowned institutions and are matched with a mentor based on their career goals and interests. The Capstone project culminates in presentations or even journal articles. This model could be used as an example for future implementation in institutions across Europe and North America.

#### Education of medical specialists

We also need to develop training opportunities for established MDs, medical specialists, to promote timely integration of systems medicine topics into the clinical practice. This is a more demanding task, since MDs have limited time available for education and training. However, experience shows that they want to gain this knowledge once they see the benefits for their patients, such as better diagnostics and treatments. To reach this target group, a variety of lifelong education possibilities has to be offered, such as systems medicine meetings, expert guided workshops and summer schools, targeted lecture series, etc. The MDs could better be attracted if the courses are accredited with the Continuous Medical Education credits, which are required for maintaining the practitioner license in several European countries.

## Strengths, Weaknesses, Opportunities, and Threats Analysis

**FIG. 5.**
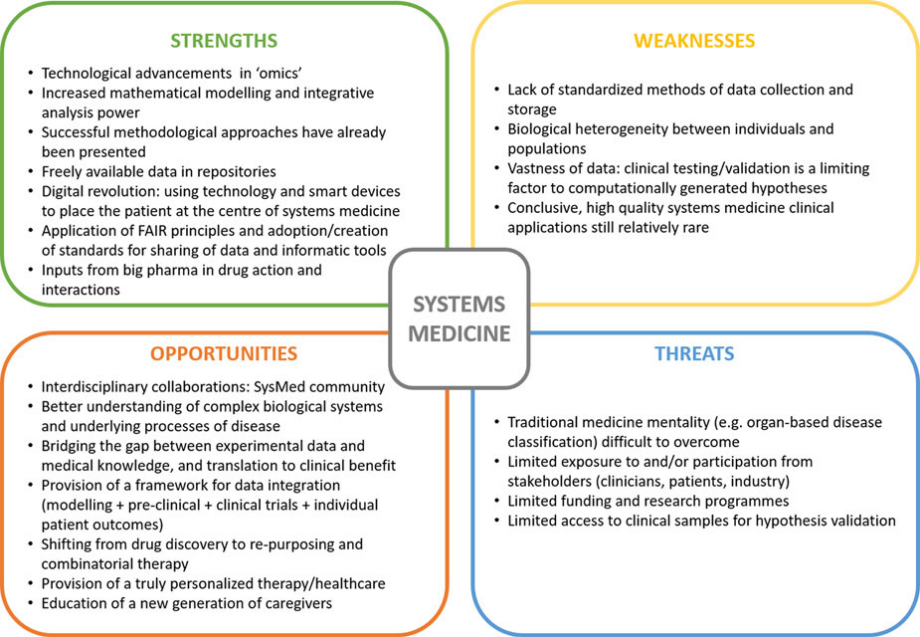
Strengths, weaknesses, opportunities, and threats analysis for network and systems medicine.^150^

To outline the different important issues for network and systems medicine, a strengths, weaknesses, opportunities, and threats analysis (Fig. 5) was performed. This thorough analysis is key for the design and development of a strategic plan that would contribute to the implementation of systems medicine in a wide spectrum of clinical applications within precision health care. In terms of strengths, that is, the innate advantages of systems medicine, the availability of multidimensional data and computational tools are important and solid elements for the field, as well as the input of the big pharma industry for applications. However, the lack of standardization in methods and data storage, as well as interindividual variability and populations limit hypothesis generations and clinical applications.

Nonetheless, major opportunities were identified: The development of multidisciplinary communities and collaborations will result in a rapid advance in knowledge and translation that should be strengthened with education, which will finally lead to a truly personalized health care system. Lastly, prompt actions will be necessary to overcome the tradition and way of thinking in medicine to reinforce participation of stakeholders and funding agencies.

## Concluding Remarks and Outlook

A considerable number of obstacles still need to be overcome for more profitable and successful implementation of practical systems medicine in the clinical setting.

Among these issues, with no claim of completeness, we include the fact that a high number of scientific hypotheses can be generated *via* systems medicine methods, and that corresponding reliable testing and solid validations—essential before safe clinical practice—are still lacking. This is mainly due to the limitation of resources to test many of such hypotheses. A comprehensive validation practice should ultimately steer toward the adoption of certified, harmonized, and partly machine-operated workflows and protocols, finally capable and designed to function in dynamic clinical contexts.

Another problem concerns the vast imbalance of systems biology studies that still focus on smaller biological systems, over the systems medicine ones, targeting larger systems/whole organisms. Along with this, there is also the interrelated difficulty to scale up to the whole organism tier, clearly necessary in the clinical practice, due to the intrinsic limits of the conclusions related to the narrow experimental/biological context (e.g., gene regulatory networks acting in cellular processes, signaling pathways analysis, etc., whose analyses often provide views that are too limited to be relevant in the clinic).

Several issues occur when collecting supporting, comprehensible, and secured (another buzzword itself, nowadays) datasets in clinical settings. Indeed, the type, arrangement, and nature of medical and clinical data have their peculiar production methods, schemas, ontologies, standards, complexity, and access limits, which often conflict with the requirement and the complications to tie unambiguously such clinical data with the clinical samples.

An often-overlooked consideration resides in the circumstance that *in silico* methods, models, and research outcomes should not be excessively complicated or abstruse to MDs, personnel, and policymakers. About this point, it may be relevant to refer here to the problem of AI, that is, machine and deep learning approaches, used to perform predictive analyses in the clinical practice. It is well known that AI algorithms often work and produce results as “black box,” that is, for its nature, it conceals the relationship and the importance of a set of data features from the output, which should finally represent the biological/medical interpretation. This is mainly because such correlations are multidimensional and not reducible enough to be easily grasped by the human mind (which is exactly why AI is used). This peculiarity of AI methods can often hamper or delay the deployment of predictive models, because humans simply do not understand, and thus trust them.^145^ Relevant efforts are being made to overcome this issue by providing interpretable ML methodologies that are capable of balancing accuracy, human interpretability, and, last but not least, computational viability.^146,147^

As a final consideration, it is now clear that the structure of the health care system will have to adjust radically to be able to run with highly interdisciplinary crews, operating daily with multi-omics, multisource data, large-scale databases and storage facilities, complex analytical processes, and—clearly—effective managerial and organizational frameworks. Such practices call for tailored education programs and continuous, complimentary training for hospital personnel as well as for systemic scientists.^36,148,149^

## Notes

**Cite this article as:** Comte B, Baumbach J, Benis A, Basílio J, Debeljak N, Flobak Å, Franken C, Harel N, He F, Kuiper M, Méndez Pérez JA, Pujos-Guillot E, Režen T, Rozman D, Schmid JA, Scerri J, Tieri P, Van Steen K, Vasudevan S, Watterson S, Schmidt HHHW (2020) Network and systems medicine: position paper of the european collaboration on science and technology action on open multiscale systems medicine, *Network and Systems Medicine* 3:1, 67–90, DOI: 10.1089/nsm.2020.0004.

## Abbreviations Used

3D: three-dimensional
AI: artificial intelligence
API: active pharmaceutical ingredient
CASyM: Coordinated Actions Systems Medicine
EASyM: European Association for Systems Medicine
EU: European Union
FAIR: findable, applicable, interoperable, and reusable
GMP: guanosine monophosphate
GWAS: genome-wide association study
HMO: Healthcare Management Organization
KEGG: Kyoto Encyclopedia of Genes and Genomes
MDs: medical doctors
MIASE: minimum information about a simulation experiment
ML: machine learning
NNT: numbers needed to treat
SBGN: systems biology graphical notation
SBML: systems biology markup language

## Notes

^*^ For details see https://sbgn.github.io

^†^ For details see http://sbml.org

^‡^ https://github.com/druglogics/cascade

## References

[1] Loscalzo J Personalized cardiovascular medicine and drug development. Circulation. 2012;125:638–645 22294708 10.1161/CIRCULATIONAHA.111.089243 PMC3273851

[2] Nosengo N Can you teach old drugs new tricks? Nature. 2016;534:314–316 27306171 10.1038/534314a

[3] Wieseler B, McGauran N, Kaiser T New drugs: where did we go wrong and what can we do better? BMJ. 2019;l4340 31292109 10.1136/bmj.l4340

[4] Schork NJ Personalized medicine: time for one-person trials. Nature. 2015;520:609–611 25925459 10.1038/520609a

[5] Scannell JW, Blanckley A, Boldon H, et al. Diagnosing the decline in pharmaceutical R&D efficiency. Nat Rev Drug Discov. 2012;11:191–200 22378269 10.1038/nrd3681

[6] Prinz F, Schlange T, Asadullah K Believe it or not: how much can we rely on published data on potential drug targets? Nat Rev Drug Discov. 2011;10:712 21892149 10.1038/nrd3439-c1

[7] Kleinschnitz C, Mencl S, Kleikers PWM, et al. NOS knockout or inhibition but not disrupting PSD-95-NOS interaction protect against ischemic brain damage. J Cereb Blood Flow Metab. 2016;36:1508–1512 27354091 10.1177/0271678X16657094 PMC5012526

[8] Kleikers PWM, Hooijmans C, Göb E, et al. A combined pre-clinical meta-analysis and randomized confirmatory trial approach to improve data validity for therapeutic target validation. Sci Rep. 2015;5:13428 26310318 10.1038/srep13428 PMC4550831

[9] Dornas WC, Silva ME Animal models for the study of arterial hypertension. J Biosci. 2011;36:731–737 21857120 10.1007/s12038-011-9097-y

[10] Segal-Lieberman G, Rosenthal T Animal models in obesity and hypertension. Curr Hypertens Rep. 2013;15:190–195 23536127 10.1007/s11906-013-0338-3

[11] Kleinschnitz C, Fluri F, Schuhmann M Animal models of ischemic stroke and their application in clinical research. Drug Des Dev Ther. 2015;9:3445 10.2147/DDDT.S56071 PMC4494187 26170628

[12] Shanks N, Greek R, Greek J Are animal models predictive for humans? Philos Ethics Humanit Med. 2009;4:2 19146696 10.1186/1747-5341-4-2 PMC2642860

[13] O'Collins VE, Macleod MR, Donnan GA, et al. 1,026 Experimental treatments in acute stroke. Ann Neurol. 2006;59:467–477 16453316 10.1002/ana.20741

[14] Seok J, Shaw Warren H, Cuenca AG, et al. Genomic responses in mouse models poorly mimic human inflammatory diseases. Proc Natl Acad Sci U S A. 2013;110:3507–3512 23401516 10.1073/pnas.1222878110 PMC3587220

[15] Vallance P An audience with Patrick Vallance. Nat Rev Drug Discov. 2010;9:834–834 21030993 10.1038/nrd3307

[16] Root AA, Smeeth L NNTs and NNHs: handle with care. Br J Gen Pract. 2017;67:133–133 28232359 10.3399/bjgp17X689797 PMC5325651

[17] Contopoulos-Ioannidis DG, Ntzani EE, Ioannidis JPA Translation of highly promising basic science research into clinical applications. Am J Med. 2003;114:477–484 12731504 10.1016/s0002-9343(03)00013-5

[18] Menche J, Sharma A, Kitsak M, et al. Uncovering disease-disease relationships through the incomplete interactome. Science. 2015;347:1257601 25700523 10.1126/science.1257601 PMC4435741

[19] Kiel C, Lastrucci C, Luthert PJ, et al. Simple and complex retinal dystrophies are associated with profoundly different disease networks. Sci Rep. 2017;7:41835 28139756 10.1038/srep41835 PMC5282568

[20] Sanchez-Vega F, Mina M, Armenia J, et al. Abstract 3302: the molecular landscape of oncogenic signaling pathways in The Cancer Genome Atlas. Bioinform Syst Biol. 2018; [Epub ahead of print]; DOI: 10.1158/1538-7445.am2018-3302 PMC6070353 29625050

[21] Casas AI, Hassan AA, Larsen SJ, et al. From single drug targets to synergistic network pharmacology in ischemic stroke. Proc Natl Acad Sci U S A. 2019;116:7129–7136 30894481 10.1073/pnas.1820799116 PMC6452748

[22] Laifenfeld D, Drubin DA, Catlett NL, et al. Early patient stratification and predictive biomarkers in drug discovery and development. Adv Exp Med Biol. 2012;645–653 10.1007/978-1-4419-7210-1_38 22161357

[23] Carrigan P, Krahn T Impact of biomarkers on personalized medicine. Handb Exp Pharmacol. 2016;232:285–311 26330258 10.1007/164_2015_24

[24] Liu R, Wang X, Aihara K, et al. Early diagnosis of complex diseases by molecular biomarkers, network biomarkers, and dynamical network biomarkers. Med Res Rev. 2014;34:455–478 23775602 10.1002/med.21293

[25] Hopkins AL Network pharmacology: the next paradigm in drug discovery. Nat Chem Biol. 2008;4:682–690 18936753 10.1038/nchembio.118

[26] Hood L, Flores M A personal view on systems medicine and the emergence of proactive P4 medicine: predictive, preventive, personalized and participatory. N Biotechnol. 2012;29:613–624 22450380 10.1016/j.nbt.2012.03.004

[27] Auffray C, Chen Z, Hood L Systems medicine: the future of medical genomics and healthcare. Genome Med. 2009;1:2 19348689 10.1186/gm2 PMC2651587

[28] Nookaew I Network Biology. Sweden: Springer 2017

[29] Ma'ayan A Introduction to network analysis in systems biology. Sci Signal. 2011;4:tr5 21917719 10.1126/scisignal.2001965 PMC3196357

[30] Kitano H Systems biology: a brief overview. Science. 2002;295:1662–1664 11872829 10.1126/science.1069492

[31] Vidal M, Cusick ME, Barabási A-L Interactome networks and human disease. Cell. 2011;144:986–998 21414488 10.1016/j.cell.2011.02.016 PMC3102045

[32] Chuang H-Y, Lee E, Liu Y-T, et al. Network-based classification of breast cancer metastasis. Mol Syst Biol. 2007;3:140 17940530 10.1038/msb4100180 PMC2063581

[33] Berlin R, Gruen R, Best J Systems medicine disease: disease classification and scalability beyond networks and boundary conditions. Front Bioeng Biotechnol. 2018;6:112 30131956 10.3389/fbioe.2018.00112 PMC6090066

[34] Hansen J, Iyengar R Computation as the mechanistic bridge between precision medicine and systems therapeutics. Clin Pharmacol Ther. 2013;93:117–128 23212109 10.1038/clpt.2012.199 PMC3963513

[35] Delhalle S, Bode SFN, Balling R, et al. A roadmap towards personalized immunology. NPJ Syst Biol Appl. 2018;4:9 29423275 10.1038/s41540-017-0045-9 PMC5802799

[36] Flores M, Glusman G, Brogaard K, et al. P4 medicine: how systems medicine will transform the healthcare sector and society. Per Med. 2013;10:565–576 25342952 10.2217/PME.13.57 PMC4204402

[37] Furman D, Campisi J, Verdin E, et al. Chronic inflammation in the etiology of disease across the life span. Nat Med. 2019;25:1822–1832 31806905 10.1038/s41591-019-0675-0 PMC7147972

[38] Renzi C, Kaushal A, Emery J, et al. Comorbid chronic diseases and cancer diagnosis: disease-specific effects and underlying mechanisms. Nat Rev Clin Oncol. 2019;16:746–761 31350467 10.1038/s41571-019-0249-6

[39] Targher G, Lonardo A, Byrne CD Nonalcoholic fatty liver disease and chronic vascular complications of diabetes mellitus. Nat Rev Endocrinol. 2018;14:99–114 29286050 10.1038/nrendo.2017.173

[40] Wafi A, Mirnezami R Translational–omics: future potential and current challenges in precision medicine. Methods. 2018;151:3–11 29792918 10.1016/j.ymeth.2018.05.009

[41] Naik A, Košir R, Rozman D Genomic aspects of NAFLD pathogenesis. Genomics. 2013;102:84–95 23545492 10.1016/j.ygeno.2013.03.007

[42] Lorbek G, Perše M, Jeruc J, et al. Lessons from hepatocyte-specific Cyp51 knockout mice: impaired cholesterol synthesis leads to oval cell-driven liver injury. Sci Rep. 2015;5:8777 25739789 10.1038/srep08777 PMC4350092

[43] Cvitanović Tomaš T, Urlep Ž, Moškon M, et al. LiverSex computational model: sexual aspects in hepatic metabolism and abnormalities. Front Physiol. 2018;9:360 29706895 10.3389/fphys.2018.00360 PMC5907313

[44] Micheel CM, Nass SJ, Omenn GS, et al. Evolution of Translational Omics: Lessons Learned and the Path Forward Washington, DC: National Academies Press 2012 24872966

[45] Nicholson JK, Lindon JC, Holmes E “Metabonomics”: understanding the metabolic responses of living systems to pathophysiological stimuli via multivariate statistical analysis of biological NMR spectroscopic data. Xenobiotica. 1999;29:1181–1189 10598751 10.1080/004982599238047

[46] Fiehn O, Kopka J, Dörmann P, et al. Metabolite profiling for plant functional genomics. Nat Biotechnol. 2000;18:1157–1161 11062433 10.1038/81137

[47] Mamas M, Dunn WB, Neyses L, et al. The role of metabolites and metabolomics in clinically applicable biomarkers of disease. Arch Toxicol. 2011;85:5–17 20953584 10.1007/s00204-010-0609-6

[48] Lindon JC, Nicholson JK The emergent role of metabolic phenotyping in dynamic patient stratification. Expert Opin Drug Metab Toxicol. 2014;10:915–919 24905565 10.1517/17425255.2014.922954

[49] Zhang A-H, Qiu S, Xu H-Y, et al. Metabolomics in diabetes. Clin Chim Acta. 2014;429:106–110 24321733 10.1016/j.cca.2013.11.037

[50] Wild CP The exposome: from concept to utility. Int J Epidemiol. 2012;41:24–32 22296988 10.1093/ije/dyr236

[51] Shaffer M, Armstrong AJS, Phelan VV, et al. Microbiome and metabolome data integration provides insight into health and disease. Transl Res. 2017;189:51–64 28764956 10.1016/j.trsl.2017.07.001 PMC5659916

[52] Shoaie S, Nielsen J Elucidating the interactions between the human gut microbiota and its host through metabolic modeling. Front Genet. 2014;5:86 24795748 10.3389/fgene.2014.00086 PMC4000997

[53] Greenblum S, Chiu H-C, Levy R, et al. Towards a predictive systems-level model of the human microbiome: progress, challenges, and opportunities. Curr Opin Biotechnol. 2013;24:810–820 23623295 10.1016/j.copbio.2013.04.001 PMC3732493

[54] Hood L, Heath JR, Phelps ME, et al. Systems biology and new technologies enable predictive and preventative medicine. Science. 2004;306:640–643 15499008 10.1126/science.1104635

[55] Galetsi P, Katsaliaki K, Kumar S Values, challenges and future directions of big data analytics in healthcare: a systematic review. Soc Sci Med. 2019;241:112533 31585681 10.1016/j.socscimed.2019.112533

[56] Laenkholm A-V, Jensen M-B, Eriksen JO, et al. PAM50 risk of recurrence score predicts 10-year distant recurrence in a comprehensive Danish cohort of postmenopausal women allocated to 5 years of endocrine therapy for hormone receptor–positive early breast cancer. J Clin Oncol. 2018;36:735–740 29369732 10.1200/JCO.2017.74.6586

[57] Slodkowska EA, Ross JS MammaPrint^TM^ 70-gene signature: another milestone in personalized medical care for breast cancer patients. Expert Rev Mol Diagn. 2009;9:417–422 19580427 10.1586/erm.09.32

[58] Bösl A, Spitzmüller A, Jasarevic Z, et al. MammaPrint versus EndoPredict: poor correlation in disease recurrence risk classification of hormone receptor positive breast cancer. PLoS One. 2017;12:e0183458 28850621 10.1371/journal.pone.0183458 PMC5574574

[59] Walter VP, Taran F-A, Wallwiener M, et al. A high-risk 70-gene signature is not associated with the detection of tumor cell dissemination to the bone marrow. Breast Cancer Res Treat. 2018;169:305–309 29374853 10.1007/s10549-018-4679-0

[60] Weinstein JN, The Cancer Genome Atlas Research Network, Collisson EA, et al. The Cancer Genome Atlas Pan-Cancer analysis project. Nat Genet. 2013;45:1113–1120 24071849 10.1038/ng.2764 PMC3919969

[61] Zhang J, Baran J, Cros A, et al. International Cancer Genome Consortium Data Portal—a one-stop shop for cancer genomics data. Database. 2011;2011:bar026 21930502 10.1093/database/bar026 PMC3263593

[62] International Agency for Research on Cancer. International Agency for Research on Cancer Biennial Report 2004–2005. Diamond Pocket Books (P) Ltd. Switzerland: WHO Press 2006

[63] Kühling J Privacy in healthcare [in German]. Medizinrecht. 2019;37:611–622

[64] Wilkinson MD, Dumontier M, Aalbersberg IJJ, et al. The FAIR guiding principles for scientific data management and stewardship. Sci Data. 2016;3:160018 26978244 10.1038/sdata.2016.18 PMC4792175

[65] Sattler F, Wiedemann S, Muller K-R, et al. Robust and communication-efficient federated learning from non-i.i.d. data. IEEE Trans Neural Netw Learn Syst. 2019:1–14 10.1109/TNNLS.2019.2944481 31689214

[66] Fang J, Fu H, Yang G, et al. RedSync: reducing synchronization bandwidth for distributed deep learning training system. J Parallel Distr Com. 2019;133:30–39

[67] Schmidt M, Schmidt SAJ, Sandegaard JL, et al. The Danish National Patient Registry: a review of content, data quality, and research potential. Clin Epidemiol. 2015;7:449–490 26604824 10.2147/CLEP.S91125 PMC4655913

[68] Schmidt M, Schmidt SAJ, Adelborg K, et al. The Danish health care system and epidemiological research: from health care contacts to database records. Clin Epidemiol. 2019;11:563–591 31372058 10.2147/CLEP.S179083 PMC6634267

[69] Jaffe DH, Flaks-Manov N, Benis A, et al. Population-based cohort of 500 patients with Gaucher disease in Israel. BMJ Open. 2019;9:e024251 10.1136/bmjopen-2018-024251 PMC6347887 30670517

[70] Boeckhout M, Zielhuis GA, Bredenoord AL The FAIR guiding principles for data stewardship: fair enough? Eur J Hum Genet. 2018;26:931–936 29777206 10.1038/s41431-018-0160-0 PMC6018669

[71] Wittig U, Rey M, Weidemann A, et al. Data management and data enrichment for systems biology projects. J Biotechnol. 2017;261:229–237 28606610 10.1016/j.jbiotec.2017.06.007

[72] Le Novère N, Hucka M, Mi H, et al. The systems biology graphical notation. Nat Biotechnol. 2009;27:735–741 19668183 10.1038/nbt.1558

[73] Benson HE, Watterson S, Sharman JL, et al. Is systems pharmacology ready to impact upon therapy development? A study on the cholesterol biosynthesis pathway. Br J Pharmacol. 2017;174:4362–4382 28910500 10.1111/bph.14037 PMC5715582

[74] Parton A, McGilligan V, Chemaly M, et al. New models of atherosclerosis and multi-drug therapeutic interventions. Bioinformatics. 2019;35:2449–2457 30520978 10.1093/bioinformatics/bty980

[75] van Iersel MP, Villéger AC, Czauderna T, et al. Software support for SBGN maps: SBGN-ML and LibSBGN. Bioinformatics. 2012;28:2016–2021 22581176 10.1093/bioinformatics/bts270 PMC3400951

[76] Hucka M, Finney A, Sauro HM, et al. The systems biology markup language (SBML): a medium for representation and exchange of biochemical network models. Bioinformatics. 2003;19:524–531 12611808 10.1093/bioinformatics/btg015

[77] Waltemath D, Adams R, Beard DA, et al. Minimum information about a simulation experiment (MIASE). PLoS Comput Biol. 2011;7:e1001122 21552546 10.1371/journal.pcbi.1001122 PMC3084216

[78] Perkel JM Why Jupyter is data scientists' computational notebook of choice. Nature. 2018;563:145–146 30375502 10.1038/d41586-018-07196-1

[79] Boettiger C An introduction to Docker for reproducible research. ACM SIGOPS Oper Syst Rev. 2015;49:71–79

[80] Benson M Clinical implications of omics and systems medicine: focus on predictive and individualized treatment. J Intern Med. 2016;279:229–240 26891944 10.1111/joim.12412

[81] Joyce AR, Palsson BØ The model organism as a system: integrating “omics” data sets. Nat Rev Mol Cell Biol. 2006;7:198–210 16496022 10.1038/nrm1857

[82] Zhang W, Li F, Nie L Integrating multiple “omics” analysis for microbial biology: application and methodologies. Microbiology. 2010;156:287–301 19910409 10.1099/mic.0.034793-0

[83] De Keersmaecker SCJ, Thijs IMV, Vanderleyden J, et al. Integration of omics data: how well does it work for bacteria? Mol Microbiol. 2006;62:1239–1250 17040488 10.1111/j.1365-2958.2006.05453.x

[84] Hamid JS, Hu P, Roslin NM, et al. Data integration in genetics and genomics: methods and challenges. Hum Genomics Proteomics. 2009;2009:869093 20948564 10.4061/2009/869093 PMC2950414

[85] Gomez-Cabrero D, Abugessaisa I, Maier D, et al. Data integration in the era of omics: current and future challenges. BMC Syst Biol. 2014;8(Suppl 2):I1 10.1186/1752-0509-8-S2-I1 PMC4101704 25032990

[86] Zierer J, Menni C, Kastenmüller G, et al. Integration of “omics” data in aging research: from biomarkers to systems biology. Aging Cell. 2015;14:933–944 26331998 10.1111/acel.12386 PMC4693464

[87] Kint G, Fierro C, Marchal K, et al. Integration of “omics” data: does it lead to new insights into host-microbe interactions? Future Microbiol. 2010;5:313–328 20143952 10.2217/fmb.10.1

[88] Van Steen K, Moore JH How to increase our belief in discovered statistical interactions via large-scale association studies? Hum Genet. 2019;138:293–305 30840129 10.1007/s00439-019-01987-w PMC6483943

[89] Gusareva ES, Van Steen K Practical aspects of genome-wide association interaction analysis. Hum Genet. 2014;133:1343–1358 25164382 10.1007/s00439-014-1480-y

[90] Figeys D Combining different “omics” technologies to map and validate protein-protein interactions in humans. Brief Funct Genomic Proteomic. 2004;2:357–365 15163370 10.1093/bfgp/2.4.357

[91] Wachter A, Beißbarth T pwOmics: an R package for pathway-based integration of time-series omics data using public database knowledge. Bioinformatics. 2015;31:3072–3074 26002883 10.1093/bioinformatics/btv323

[92] Luscombe NM, Greenbaum D, Gerstein M What is bioinformatics? An introduction and overview. Yearb Med Inform. 2001;10:83–100 27701604

[93] Chari R, Coe BP, Wedseltoft C, et al. SIGMA2: a system for the integrative genomic multi-dimensional analysis of cancer genomes, epigenomes, and transcriptomes. BMC Bioinformatics. 2008;9:422 18840289 10.1186/1471-2105-9-422 PMC2571113

[94] Génin E, Devoto M Integration of omics data in genetic epidemiology. Hum Hered. 2015;79:109–110 26201696 10.1159/000382041

[95] López de Maturana E, Pineda S, Brand A, et al. Toward the integration of Omics data in epidemiological studies: still a “long and winding road.” Genet Epidemiol. 2016;40:558–569 27432111 10.1002/gepi.21992

[96] Cai X, Bazerque JA, Giannakis GB Inference of gene regulatory networks with sparse structural equation models exploiting genetic perturbations. PLoS Comput Biol. 2013;9:e1003068 23717196 10.1371/journal.pcbi.1003068 PMC3662697

[97] Pinu FR, Beale DJ, Paten AM, et al. Systems biology and multi-omics integration: viewpoints from the metabolomics research community. Metabolites. 2019;9:76 10.3390/metabo9040076 PMC6523452 31003499

[98] Kaever A, Landesfeind M, Feussner K, et al. Meta-analysis of pathway enrichment: combining independent and dependent omics data sets. PLoS One. 2014;9:e89297 24586671 10.1371/journal.pone.0089297 PMC3938450

[99] López de Maturana E, Alonso L, Alarcón P, et al. Challenges in the integration of omics and non-omics data. Genes (Basel). 2019;10:238 10.3390/genes10030238 PMC6471713 30897838

[100] Fouladi R. From statistical to biological interactions towards an omics-integrated MB-MDR framework. PhD Doctor in Electrical Engineering and Computer Science. Université de Liège, Liège, Belgique. Available at: https://orbi.uliege.be/handle/2268/228579 (last accessed 96, 2018)

[101] Tenenhaus M, Vinzi VE, Chatelin Y-M, et al. PLS path modeling. Comput Stat Data Anal. 2005;48:159–205

[102] Risi Imre Kondor JL Diffusion kernels on graphs and other discrete structures. In: Proceedings of the ICML. Available at: http://citeseerx.ist.psu.edu/viewdoc/summary?doi=10.1.1.128.4966 2002 (last accessed 224, 2020)

[103] Morota G, Koyama M, Rosa GJM, et al. Predicting complex traits using a diffusion kernel on genetic markers with an application to dairy cattle and wheat data. Genet Sel Evol. 2013;45:17 23763755 10.1186/1297-9686-45-17 PMC3706293

[104] Zhang Y, Hu X, Jiang X Multi-view clustering of microbiome samples by robust similarity network fusion and spectral clustering. IEEE/ACM Trans Comput Biol Bioinform. 2017;14:264–271 26513798 10.1109/TCBB.2015.2474387

[105] Wang B, Mezlini AM, Demir F, et al. Similarity network fusion for aggregating data types on a genomic scale. Nat Methods. 2014;11:333–337 24464287 10.1038/nmeth.2810

[106] Chung R-H, Kang C-Y A multi-omics data simulator for complex disease studies and its application to evaluate multi-omics data analysis methods for disease classification. Gigascience. 2019;8:giz045 31029063 10.1093/gigascience/giz045 PMC6486474

[107] Koh HWL, Fermin D, Vogel C, et al. iOmicsPASS: network-based integration of multiomics data for predictive subnetwork discovery. NPJ Syst Biol Appl. 2019;5:22 31312515 10.1038/s41540-019-0099-y PMC6616462

[108] Licata L, Lo Surdo P, Iannuccelli M, et al. SIGNOR 2.0, the SIGnaling Network Open Resource 2.0: 2019 update. Nucleic Acids Res. 2020;48:D504–D510 31665520 10.1093/nar/gkz949 PMC7145695

[109] Jassal B, Matthews L, Viteri G, et al. The reactome pathway knowledgebase. Nucleic Acids Res. 2020;48:D498–D503 31691815 10.1093/nar/gkz1031 PMC7145712

[110] Flobak Å, Baudot A, Remy E, et al. Discovery of drug synergies in gastric cancer cells predicted by logical modeling. PLoS Comput Biol. 2015;11:e1004426 26317215 10.1371/journal.pcbi.1004426 PMC4567168

[111] Flobak Å, Niederdorfer B, Nakstad VT, et al. A high-throughput drug combination screen of targeted small molecule inhibitors in cancer cell lines. Sci Data. 2019;6:237 31664030 10.1038/s41597-019-0255-7 PMC6820772

[112] Ooft SN, Weeber F, Dijkstra KK, et al. Patient-derived organoids can predict response to chemotherapy in metastatic colorectal cancer patients. Sci Transl Med. 2019;11:eaay2574 31597751 10.1126/scitranslmed.aay2574

[113] Weeber F, Ooft SN, Dijkstra KK, et al. Tumor organoids as a pre-clinical cancer model for drug discovery. Cell Chem Biol. 2017;24:1092–1100 28757181 10.1016/j.chembiol.2017.06.012

[114] Musizza B, Ribaric S Monitoring the depth of anaesthesia. Sensors. 2010;10:10896–10935 22163504 10.3390/s101210896 PMC3231065

[115] Reboso JA, Gonzalez-Cava JM, León A, et al. Closed loop administration of propofol based on a Smith predictor: a randomized controlled trial. Minerva Anestesiol. 2019;85:585–593 30394065 10.23736/S0375-9393.18.13058-6

[116] Hund HC, Rice MJ, Ehrenfeld J An evaluation of the state of neuromuscular blockade monitoring devices. J Med Syst. 2016;40:281 27787785 10.1007/s10916-016-0641-z

[117] Cowen R, Stasiowska MK, Laycock H, et al. Assessing pain objectively: the use of physiological markers. Anaesthesia. 2015;70:828–847 25772783 10.1111/anae.13018

[118] Martín-Mateos I, Méndez Pérez JA, Reboso Morales JA, et al. Adaptive pharmacokinetic and pharmacodynamic modelling to predict propofol effect using BIS-guided anesthesia. Comput Biol Med. 2016;75:173–180 27294779 10.1016/j.compbiomed.2016.06.007

[119] Mathis MR, Kheterpal S, Najarian K Artificial intelligence for anesthesia. Anesthesiology. 2018;129:619–622 30080689 10.1097/ALN.0000000000002384 PMC6148374

[120] Marrero A, Méndez JA, Reboso JA, et al. Adaptive fuzzy modeling of the hypnotic process in anesthesia. J Clin Monit Comput. 2017;31:319–330 27072987 10.1007/s10877-016-9868-y

[121] Eskandari N, van Heusden K, Dumont GA Extended habituating model predictive control of propofol and remifentanil anesthesia. Biomed Signal Process Control. 2020;55:101656

[122] Moore BL, Quasny TM, Doufas AG Reinforcement learning versus proportional–integral–derivative control of hypnosis in a simulated intraoperative patient. Anesth Analg. 2011;112:350–359 21156973 10.1213/ANE.0b013e318202cb7c

[123] Eshghi N, Aliyari M, Teshnehlab M Anesthesia control based on intelligent controllers. 2009 3rd International Conference on Bioinformatics and Biomedical Engineering. Beijing, China. 2009 [Epub ahead of print]; DOI: 10.1109/icbbe.2009.5162370

[124] Gonzalez-Cava JM, Reboso JA, Casteleiro-Roca JL, et al. A novel fuzzy algorithm to introduce new variables in the drug supply decision-making process in medicine. Complexity. 2018;2018:1–15

[125] Afsana, Jain V, Haider N, et al. 3D printing in personalized drug delivery. Curr Pharm Des. 2018;24:5062–5071 30767736 10.2174/1381612825666190215122208

[126] Wang G, Dunbrack RL PISCES: a protein sequence culling server. Bioinformatics. 2003;19:1589–1591 12912846 10.1093/bioinformatics/btg224

[127] Chartier M, Morency L-P, Zylber MI, et al. Large-scale detection of drug off-targets: hypotheses for drug repurposing and understanding side-effects. BMC Pharmacol Toxicol. 2017;18:18 28449705 10.1186/s40360-017-0128-7 PMC5408384

[128] Axén I, Bodin L, Bergström G, et al. Clustering patients on the basis of their individual course of low back pain over a six month period. BMC Musculoskelet Disord. 2011;12:99 21586117 10.1186/1471-2474-12-99 PMC3125255

[129] Rai A, Chen L, Pye J, et al. Understanding determinants of consumer mobile health usage intentions, assimilation, and channel preferences. J Med Internet Res. 2013;15:e149 23912839 10.2196/jmir.2635 PMC3742412

[130] Hoffman AS, Volk RJ, Saarimaki A, et al. Delivering patient decision aids on the Internet: definitions, theories, current evidence, and emerging research areas. BMC Med Inform Decis Mak. 2013;13:S13 24625064 10.1186/1472-6947-13-S2-S13 PMC4043476

[131] Beck F, Richard J-B, Nguyen-Thanh V, et al. Use of the internet as a health information resource among French Young adults: results from a nationally representative survey. J Med Internet Res. 2014;16:e128 24824164 10.2196/jmir.2934 PMC4051740

[132] Moick M, Terlutter R Physicians' motives for professional internet use and differences in attitudes toward the internet-informed patient, physician–patient communication, and prescribing behavior. Medicine 2.0. 2012;1:e2 25075230 10.2196/med20.1996 PMC4084769

[133] Kritz M, Gschwandtner M, Stefanov V, et al. Utilization and perceived problems of online medical resources and search tools among different groups of European physicians. J Med Internet Res. 2013;15:e122 23803299 10.2196/jmir.2436 PMC3713956

[134] Dugdale DC, Epstein R, Pantilat SZ Time and the patient-physician relationship. J Gen Intern Med. 1999;14:S34–S40 9933493 10.1046/j.1525-1497.1999.00263.x PMC1496869

[135] Weiner JP Doctor–patient communication in the e-health era. Isr J Health Policy Res. 2012;1:33 22929000 10.1186/2045-4015-1-33 PMC3461429

[136] Peleg R, Avdalimov A, Freud T Providing cell phone numbers and email addresses to patients: the physician's perspective. BMC Res Notes. 2011;4:76 21426591 10.1186/1756-0500-4-76 PMC3076270

[137] Peleg R, Nazarenko E Providing cell phone numbers and e-mail addresses to patients: the patient's perspective, a cross sectional study. Isr J Health Policy Res. 2012;1:32 22929801 10.1186/2045-4015-1-32 PMC3441808

[138] Henao R, Murray J, Ginsburg G, et al. Patient clustering with uncoded text in electronic medical records. AMIA Annu Symp Proc. 2013;2013:592–599 24551361 PMC3900202

[139] Sewitch MJ, Leffondré K, Dobkin PL Clustering patients according to health perceptions. J Psychosom Res. 2004;56:323–332 15046970 10.1016/S0022-3999(03)00508-7

[140] Benis A, Harel N, Barkan R, et al. Identification and description of healthcare customer communication patterns among individuals with diabetes in clalit health services: a retrospective database study. Stud Health Technol Inform. 2017;244:18–22 29039369

[141] Benis A, Barak Barkan R, Sela T, et al. Communication behavior changes between patients with diabetes and healthcare providers over 9 years. J Med Internet Res. [Epub ahead of print]; DOI: 10.2196/17186 PMC7448191 32648555

[142] Nelson EC, Dixon-Woods M, Batalden PB, et al. Patient focused registries can improve health, care, and science. BMJ. 2016;354:i3319 27370543 10.1136/bmj.i3319 PMC5367618

[143] Nelson EC, Eftimovska E, Lind C, et al. Patient reported outcome measures in practice. BMJ. 2015;350:g7818 25670183 10.1136/bmj.g7818

[144] Rozman D, Acimovic J, Schmeck B Training in systems approaches for the next generation of life scientists and medical doctors. Methods Mol Biol. 2016;1386:73–86 26677180 10.1007/978-1-4939-3283-2_5

[145] Camacho DM, Collins KM, Powers RK, et al. Next-generation machine learning for biological networks. Cell. 2018;173:1581–1592 29887378 10.1016/j.cell.2018.05.015

[146] Lakkaraju H, Bach SH, Jure L Interpretable decision sets: a joint framework for description and prediction. KDD. 2016;2016:1675–1684 27853627 10.1145/2939672.2939874 PMC5108651

[147] Yu MK, Ma J, Fisher J, et al. Visible machine learning for biomedicine. Cell. 2018;173:1562–1565 29906441 10.1016/j.cell.2018.05.056 PMC6483071

[148] Hood L Systems biology and p4 medicine: past, present, and future. Rambam Maimonides Med J. 2013;4:e0012 23908862 10.5041/RMMJ.10112 PMC3678833

[149] Noell G, Faner R, Agustí A From systems biology to P4 medicine: applications in respiratory medicine. Eur Respir Rev. 2018;27:170110 29436404 10.1183/16000617.0110-2017 PMC9489012

[150] Auffray C, Balling R, Benson M, et al. From Systems Biology to Systems Medicine. Workshop Report. European Commission, DG Research, Directorate of Health. Brussels, Belgium, June 14–15, 2010

